# Relationships between Th1 or Th2 iNKT Cell Activity and Structures of CD1d-Antigen Complexes: Meta-analysis of CD1d-Glycolipids Dynamics Simulations

**DOI:** 10.1371/journal.pcbi.1003902

**Published:** 2014-11-06

**Authors:** Xavier Laurent, Nicolas Renault, Amaury Farce, Philippe Chavatte, Eric Hénon

**Affiliations:** 1 Intestinal Biotech Development, Faculté de Médecine, Lille, France; 2 Laboratoire de Chimie Thérapeutique EA4481, Université Lille 2, Lille, France; 3 Institut de Chimie Pharmaceutique Albert Lespagnol EA4481, Université Lille 2, Lille, France; 4 Institut de Chimie Moléculaire de Reims UMR CNRS 7312, University of Reims Champagne-Ardenne, Reims, France; Max Planck Institute for Biophysical Chemistry, Germany

## Abstract

A number of potentially bioactive molecules can be found in nature. In particular, marine organisms are a valuable source of bioactive compounds. The activity of an α-galactosylceramide was first discovered in 1993 via screening of a Japanese marine sponge (*Agelas mauritanius*). Very rapidly, a synthetic glycololipid analogue of this natural molecule was discovered, called KRN7000. Associated with the CD1d protein, this α-galactosylceramide **1** (KRN7000) interacts with the T-cell antigen receptor to form a ternary complex that yields T helper (Th) 1 and Th2 responses with opposing effects. In our work, we carried out molecular dynamics simulations (11.5 µs in total) involving eight different ligands (conducted in triplicate) in an effort to find out correlation at the molecular level, if any, between chemical modulation of **1** and the orientation of the known biological response, Th1 or Th2. Comparative investigations of human versus mouse and Th1 versus Th2 data have been carried out. A large set of analysis tools was employed including free energy landscapes. One major result is the identification of a specific conformational state of the sugar polar head, which could be correlated, in the present study, to the biological Th2 biased response. These theoretical tools provide a structural basis for predicting the very different dynamical behaviors of α-glycosphingolipids in CD1d and might aid in the future design of new analogues of **1**.

## Author Summary

To modulate the natural immune response toward aggressive (Th1) or protective (Th2) profiles remains a difficult challenge, but can also offer great therapeutic opportunities, particularly for the treatment of cancer or auto-immune diseases. It has been demonstrated that a particular type of cells, named invariant Natural Killer T (iNKT) cells, are able to induce both protective and aggressive response profiles, depending on the antigen that is presented to it by CD1d proteins. Since this discovery, efforts have been made to find synthetic compounds that would selectively induce Th1 or Th2 immune response. KRN7000 was the first to selectively induce a Th1 response, and for two decades many analogues of this compound were synthesized. Some of them effectively induce Th1- or Th2-biased responses. But, unfortunately, the Th1/Th2 selectivity mechanism remains unclear. That is the reason why we have undertaken large-scale molecular modeling (molecular dynamics) simulations of various CD1d-ligand systems with the aim to find out correlation between chemical modulation and the orientation of the biological response for a variety of known ligands.

## Introduction

**Figure 1:**
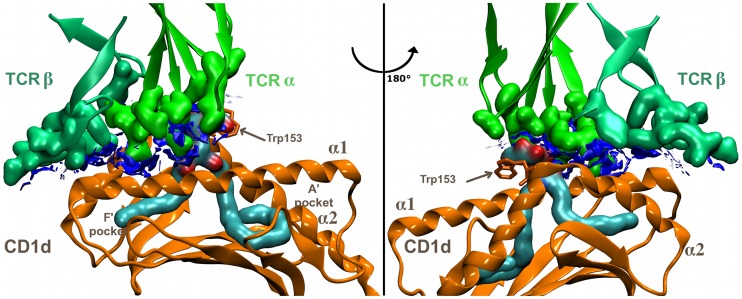
(CD1d-1)-TCR footprint. Reduced density gradient isosurfaces (blue, s = 0.6) provide a meaningful visualization of non-covalent interactions (NCI) as wide regions of molecular space in the TCR-CD1d binding domain; for the sake of clarity, these surfaces are not colored according to the strength of the interaction.

Compound **1**, [1], [2] also referred to as α-GalCer, is a synthetic glycolipid that has shown promising bioactivity against diverse pathologies (atherosclerosis, malaria, auto-immune diseases…). [3]–[7] This compound is presented to the iNKT cells via a MHC class 1-like protein, named CD1d, associated to β2-microglobulin. **1** can be readily loaded onto both mouse and human CD1d. The resulting binary complex is carried by Antigen-Presenting Cells (APCs) such as dendritic cells and macrophages. Upon recognition of the CD1d-glycolipid complex by the T Cell Receptor (TCR) (Figure 1), the iNKT cells rapidly initiate response that leads to a release of cytokines implied in Th1 (interferon-γ, IFN-γ) and Th2 (Interleukin-4, IL-4) immune response profiles, which yields opposing results from a medicinal point of view. Much research has been focused on being able to control the cascade by attempting to bias the cytokine release profile Th1/Th2. Many biological and synthetic studies have been undertaken by a variety of research groups aimed at understanding the mechanism of **1** recognition in regards to both CD1d and TCR proteins with the hope of finding novel analogues with improved biological response (magnitude and profile selectivity of the iNKT cell stimulation). [8]–[10] But, for the time being, the relationship between glycolipid pharmacomodulation and cytokine polarization is not completely understood. However, principles were established from earlier studies, based on the stability of the CD1d-ligand-TCR trimolecular complex. Mc Carthy et al. demonstrated experimentally that modifications of the lipid chain buried in the F′ channel of human CD1d molecules (Figure 1) can modulate the TCR affinity. [11] However, they also showed that even though the length of the acyl chain controls the stability of the binary complex it does not automatically affect the CD1d-glycolopid complex affinity to TCR. In order to affect the binding affinity for TCR, it seems that a ligand chemical variation must additionally induce conformational changes of CD1d, which propagate to the TCR recognition surface. For example, these authors demonstrated that the incomplete occupation of the human CD1d F′ channel by the chain-shortened analogue **2** (OCH) [12] results in a less stable binary complex but also suggested that this causes conformational differences at the TCR recognition surface. In other respects, Porcelli et al. [13] have shown that the sugar head group of the ligand contacts the TCR in the initial phase whereas CD1d contacts with the TCR contribute to the stability of the whole complex. Since the IL-4 production was shown to require shorter TCR stimulation than IFN-γ, it has been thought to generate less stable CD1d-ligand complexes in order to impair the interaction at the ternary interface and then to elicit the cytokine profile toward a Th2 response. Conversely, a biased Th1 response was predicted through increasingly stable CD1d-glycolipid complexes. Hence, all attempts to design new ligands that polarize the cytokine profile were based on this principle of stability of the binary and ternary complexes, however not taking into account directly CD1d conformational changes induced by ligand modulations. Many modulations have been envisaged. Derivatives were obtained by changing at least one of the four distinct portions describing **1**: the sugar part, the osidic bond, the polar linker, and the two lipid chains. Throughout our manuscript, the term “polar head” will refer to the distinct fragment of the ligand that is (α-anomerically) linked to the ceramide, regardless of the analogue. This designates the group that protrudes out the binding groove of the CD1d, towards the TCR, in contrast to the two more deeply anchored alkyl chains.

In order to explain observed biological evaluation of analogues of **1**, molecular modeling supplements have sometimes been given by authors. However, these theoretical approaches are often limited to molecular docking or local optimizations of the ligand into the CD1d pockets. According to the huge number of degrees of freedom of **1** and its analogues such docking simulations are not appropriate to understand the mechanisms involved in such complex recognition process. Pipelier et al. [14] recently employed ab initio QM/QM′ level of theory to estimate electron withdrawing effect induced by the introduction of mono or difluoro substituent at C3. Such CPU extensive quantum mechanical study is however limited to a part only of the entire system and to a single structure. In order to explore the impact of a single-amino acid variation at position 93 of iNKT on the conformational stability of Complementarity Determining Region (CDR) 3α, Gadola et al. [15] recently carried out molecular dynamics (MD) simulations of the ternary complex. Though very interesting, several questions arise from such simulation. Can tools for measuring conformational stability of CDRs be restrained to a single C_α_-C_α_ distance probability distribution function? Will the expected conformational change be observed during a unique 10 ns simulation? Unlike experimental studies, only a few molecular modeling studies were fully dedicated to the investigation of the binary (CD1d-glycoliplid) or ternary (CD1d-glycolipid-TCR) complexes. To our knowledge only two previous studies have been fully devoted to the theoretical study of these systems. Nadas et al. [16] performed molecular dynamics of the ternary complex that allowed for the in-depth statistical and visual analysis of the H-bond network between CD1d, TCR and a set of 12 ligands during the simulation. The study was however limited to a single 3 ns simulation of a truncated complex, and tools for analysis were limited to hydrogen bond monitoring and visual inspection. In their theoretical study, E. Henon et al. [17] addressed the influence of three modulations on the dynamic behavior of the CD1d-glycolipid complex. However, only one 10 ns trajectory was produced for each of the four envisaged binary systems. In this previous study, the influence of the ligand modulations on the dynamic behavior of the CD1d-glycolipid complex was addressed by distance analysis and mainly focused on the so-called OTAN H-bond network built up from 2-OH, Thr154, Asp151, and NH. To be able to predict the strength of the Th1/Th2 polarization, very recently, De Spiegeleer et al. have presented multi-linear regression (MLR) and partial least squared (PLS) models based on a set of chemical descriptors of the ligand. [18] Though simple and easy to implement, these statistical methods partially failed to explain Th2 biased responses in vitro, and the use of numerous chemical descriptors prevents us from truly understanding the underlying correlation between chemical alterations and the cytokine-responses.

Besides, for now, no MD simulations have been performed for characterizing differences between recognition of glycolipid by human or mouse CD1d. This point is important since there may be difference between antigen recognition by mouse and human iNKT cell. Nor is there any study of the influence of spacer lipids on the conformational behavior of the protein. Actually, sometimes, the CD1d protein has got non-specific lipid into its pockets, even in presence of a ligand (for instance the shortened glycolipid **2**).

Clearly, molecular dynamics is one of the most appropriate tools to study interactions in these complexes and to examine how chemical variation can affect their properties. However, as previously explained, since the ligand binding affinity to CD1d is not systematically correlated to the affinity of TCR to the binary complex, this reduces considerably the interest of ligand binding affinity predictions (such as relative MMGB-SA calculations [19]). Moreover, relative binding free energy calculations via alchemical transformations [20] were ruled out due to the very large number of degrees of freedom in the two lipid chains in **1**. Simulating the ternary TCR-ligand-CD1d complex would be a very interesting study, but it would require still larger sampling compared with the binary system. Indeed, such a ternary complex is a very different system from the binary one. The TCR binding involves many additional interactions, compared to the binary complex, some of which stabilize the polar head at the binding interface (Phe29, Ser30 of CDR1alpha, and Gly96 of CDR3alpha). Thus, the impact of chemical alterations of the ligand involved in the ternary “lock and key” recognition might be observed but at a much larger time scale. Therefore, we have chosen another route. Since the TCR recognition process requires that the binding footprint onto CD1d to be maintained, instability of the ternary complex can occur only if this binding footprint is deteriorated. Figure 1 shows the non-covalent interactions[21], [22] at this interface in the X-ray structure of human CD1d-**1**-TCR ternary complex (PDB reference 2PO6). Any deformation of this interface may disable the interaction with TCR, or at least makes it less effective. Hence, the idea is to focus on the binary complex (CD1d-ligand) and to determine how a chemical modulation of the ligand loaded into CD1d affects the interface part of this binary complex. Since binary complex-TCR contacts involve both the ligand polar head and the α_1_/α_2_ helices of CD1d, these two portions of the complex have to be particularly monitored during the simulations. Most of the SAR approach assumes that the structure of the ligand alone contains the features responsible for its biological properties. Here, using molecular dynamics, we took another step forward by studying the propagation of conformational changes (induced by a chemical alteration) from within the binding grove to the surface of the binary complex. In other words, in our study, we consider the whole binary complex as a “ligand” in correlating biological activity to chemical space.

The reason why the TCR recognition process is altered may be due to an irreversible structural deformation of a portion of the interface or it may also result from an unusual dynamical behavior of this interface caused by abnormal fluctuations with larger amplitude for example. From MD trajectories, detecting these fluctuation deviations can be quite difficult because they can occur at different scales: small scale (hydrogen-bond, residue fluctuation, polar head rocking) or at even a larger scale (secondary structure motions). That is the reason why, beyond simple distance monitoring, employing additional appropriate analyzing tools is important to reveal such unusual dynamic behavior. The question is whether chemical modulations known to induce a Th2 profile (or Th1) will give MD simulations with similar features and readily detectable with post processing tools. This methodological and interpretative task is made more difficult since some ligands generally induce simultaneously both Th1 and Th2 responses (only a bias is experimentally observed) and also because experimental protocols that measure this bias can be quite different making it difficult to rigorously compare the Th1/Th2 bias values. Biological evaluations from different data sources can also sometimes be contradictory. [23] Furthermore, other factors such ligand solubility, biodisponibility, or stability in biological systems, may also play a role, which cannot be handled with our simple MD models.

**Figure 2:**
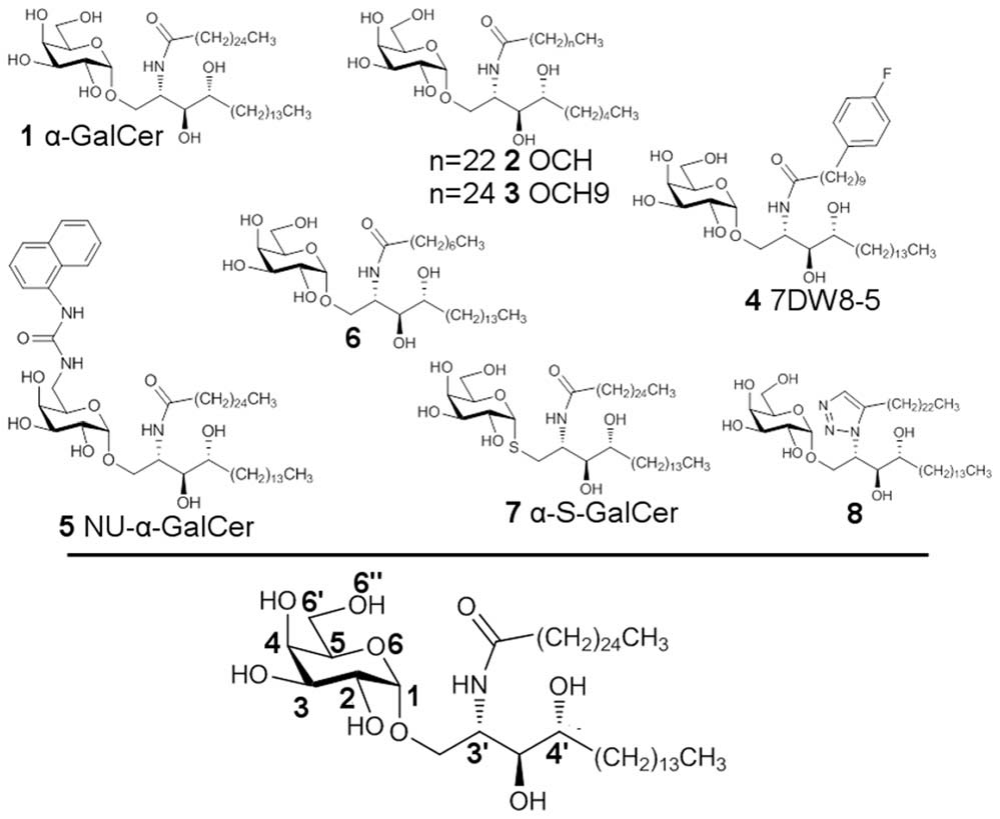
Structure and numbering of the ligands involved in the studied systems.

In our work, we carried out 48 MD simulation (11.5 µs in total) involving eight different ligands (conducted in triplicate) (Figure 2) in an effort to probe if a ligand modulation, which is known to lead to a Th2 bias, impacts on the conformational stability of the system, and how this ligand alteration is reflected in the dynamic behavior of the whole molecular structure. To what extent computational tools are able to predict a Th2 bias is very challenging for the design of new ligands. The main aim is then to test whether such a simple rule: complex instability-Th2/complex stability-Th1, is really reliable or not. From our simulations, human and mouse CD1ds are found to exhibit different structural dynamics. Moreover, specific dynamical features haven been identified, which could be correlated here to Th2-biased systems exclusively.

## Materials and Methods

### Studied systems

Overall, 16 systems have been simulated and analyzed (Table 1, Figure 2). Whenever it was possible, the short name chosen by the authors for the ligand has been followed. But additionally, the prefixes “H” or “M” have been inserted and stand for human or mouse, respectively. This set has been established in order to allow three types of comparison: human CD1d against mouse CD1d simulations, Th1 versus Th2 response and simulations with or without spacer lipid. The 2PO6 and 3SDA PDB structures[24], [25] were used for the human and mouse CD1ds, respectively.

**Table 1:** List of 16 various systems involving human or mouse CD1d and up to eight different ligands.

System name	N°	Ligand	CD1d PDB	Organism	Filler Lipid	Bias	Ref.
H_aGAL	Sy1	**1**	2PO6	Human	No	Reference	[44], [45]
M_aGAL	Sy2		3SDA	Mouse			[2]
H_OCH	Sy3	**2**	2PO6	Human	PDB 3ARB	Th2	[12]
M_OCH	Sy4		3SDA	Mouse			[12], [46]
H_OCH9	Sy5	**3**	2PO6	Human	No	Th2	[11]
H_7DW	Sy6	**4**	2PO6	Human	No	Th1	[26]
M_7DW	Sy7		3SDA	Mouse			[26]
H_NUaGAL	Sy8	**5**	2PO6	Human	No	Th1	[47]
M_NUaGAL	Sy9		3SDA	Mouse			[30], [47]
H_GOF	Sy10	**6**	2PO6	Human	from 1Z5L	Th2	[29]
M_GOF	Sy11		3SDA	Mouse		Th2	[29]
H_SaGAL	Sy12	**7**	2PO6	Human	No	Th2	[32]
M_SaGAL	Sy13		3SDA	Mouse		Inactive	[31]
M_AZOL	Sy14	**8**	3SDA	Mouse	No	Th2	[33]
H_CD1d	Sy15	Lipid free	2PO6	Human	No	NA	
H_LIP	Sy16	Two lipids	2PO6	Human	PDB 3ARB&1Z5L	NA	

Seven analogues of **1** have been chosen so as to account for modifications on the four portions of the glycolipid: the sugar part, the osidic bond, the polar linker, and the two lipid chains (Figure 2). The ligand **4** (7DW8-5) has got a phenyl group at the end of the shortened acyl chain. This analogue was shown to induce a Th1 biased response against human iNKT cells in vitro with binding affinity to human and mouse CD1d molecules. [26] The H_LIP designates simulations where the ligand has been replaced by two free lipid chains in the pockets F′ (C_12_) and A′ (C_16_) of the CD1d protein, thus, with no polar head present. These lipid structures have been taken from PDB files 3ARB [27] and 1Z5L. [28] The analogue **6** [29] has its acyl lipid chain truncated to eight carbon atoms and a Th2 polarization response of iNKT cells has been determined for this glycolipid. The **5** (NU-α-GalCer) ligand presents an ureido-naphtyl-group at position 6″ and exhibits pronounced Th1-biased cytokine production. [30] No interaction is observed between this ureido-naphtyl-group and the TCR part in the X-ray structure (PDB 3QUZ). The analogue **2** is a glycolipid with a shortened sphingosin chain (9 carbon length) and was found to induce a pronounced Th2-biased cytokine release compared to **1**. The molecule **3** (OCH9) is almost identical to **2**, only differing from it by the addition of two methylene groups on the acyl chain. In the study by Mac Carthy et al. [11] the ratio of IL-4/IFN-γ found for this compound showed clearly a Th2-bias compared to the ratio obtained for **1**. **7** (α-S-GalCer) is a thioglycoside analogue of **1** and did not activate murine iNKT cells in vivo. [23], [31], [32] But, there are conflicting studies for the biological evaluation of **7** for human iNKT cells that is predicted to elicit a preferential Th2-biased response [32] or no real bias compared to **1**. [23] Substitution of the amide function by a 1,2,3- triazole group, compound **8**, induces Th2 cytokine production. [33] Finally, the human CD1d alone has been simulated (H_CD1d) without any ligand and free lipid chains in its pockets.

### Molecular dynamics

A crystal structure is not available for each of the 16 studied systems. For some of them, the X-ray data are available but unfortunately they leave some protein structures incomplete. That is the reason why we selected only two CD1d structures: 2PO6 (human CD1d) and 3SDA (mouse CD1d) with full CD1d structures. Then, starting from the geometry of the KRN7000 ligand from PDB file 2PO6 we generated the OCH9, AZOL, SaGAL, and 7DW analogues. For this, the program molden [34] was used to achieve chemical alterations. Truncating the sphingosine chain to obtain the OCH9 compound was straightforward. Using the Z-matrix editor of Molden allowed us substituting the amide function by a 1,2,3-triazole group (AZOL compound), substituting an oxygen atom by a sulfur atom (SaGAL) and adding a phenyl group at the end of the shortened acyl chain (analogue 7DW). In this way, the derived analogues fitted naturally into the hydrophobic pockets of human CD1d (2PO6). In order for these analogues to fit also in the mouse (3SDA) CD1d we performed the superposition of the 3SDA structure on the 2PO6 one (using sequence alignment). The geometries of the analogues GOF, NUaGAL and OCH were taken from X-ray structures 1Z5L, 3QUZ and 3ARB, respectively. These protein structures were previously aligned to the 2PO6 one in order for the associated ligands to fit in the two binding pockets. Concerning the NUaGAL ligand, we had to modify the ureido-naphtyl group position in order to avoid steric clash with Trp153 in human CD1d. All these generated structures were then minimized in a first step before molecular dynamics simulations. Whenever a spacer lipid was added, it was taken from crystal structures 3ARB (spacer lipid simultaneously present with the sphingosine chain) and 1Z5L (spacer lipid simultaneously present with the acyl chain).

In the primary sequence of CD1d, the mouse protein contains an insertion of two residues between residues 89 and 90 of human CD1d. This does not concern the binding domain. The preparation of the protein (disulfide bond linkages, protonation state of ionisable side chains) was achieved using the same protocol as in our previous study. [17] In particular, as specified in the X-Ray structures, three disulfide covalent bonds were set between cysteine residues. We employed the package program xLeap of the AMBER11 package [35] to add the hydrogen atoms to the protein structure. The Propka [36] application was used to examine the protonation state of ionizable side chains with a focus on residues asparte, cysteine, histidine and glutamate in proximity to the ligand. No protonation state change was required compared to the state proposed by xLeap. Counterions (Na^+^) were added such that to neutralize the unit system. The ff99SB force field was used for the protein. The general amber force field GAFF was used in conjunction with the antechamber program to describe the ligands. [37] The respgen procedure was employed to derive atomic charges from HF/6–31G* electrostatic potential calculations obtained with the Gaussian package [38]. The ligand and protein CD1d were fully explicitly solvated in a truncated octahedral box using TIP3P water molecules with a buffer distance of 7.0 Å and under periodic boundary conditions. We have carefully checked that the initial water molecules buffer remains during our simulations, and the CD1d protein does not interact with its neighbors in periodic images. The entire system consisted of about 40700 atoms (depending on the ligand).

All the dynamics calculations were carried out with the programs sander and pmemd of the AMBER11 package. The particle mesh Ewald procedure was employed to handle long-range electrostatic interactions. The default value of 8.0 Å for the non-bonded cutoff was set to calculate van der Waals and electrostatic energies. No switching function was used for the van der Waals interactions. The system was then carefully prepared. At first, the energy of the entire system was minimized with 1000 cycles of steepest descent followed by 1000 cycles of conjugate gradient minimization. This process was repeated twice. Classical Langevin NVT molecular dynamics simulations (collision frequency  = 2 ps^−1^) employed a 2 fs integration time step (bonds involving hydrogen atoms were constrained). Firstly, the water molecules were heated from 0 to 300K during 20 ps, and equilibrated for 20 ps. A force constant of 100 kcal.mol^−1^.Å^−2^ was used to restrain all other atoms during these steps. After cooling the solvent molecules to 0 K during 20 ps, this heating-equilibrating procedure was re-run twice for the entire system with no constraint and followed by 240 ns of data collection. Prior to production, in order to allow the density to equilibrate, our system has been equilibrated using the NPT ensemble (isothermal-isobaric ensemble) at 300K and 1 atm for 40 ps.

Three 240 ns trajectories have been produced in parallel for each of the 16 systems (0.72 µs each). Coordinates of the system were written every 1 ps.

### Ligand force field assessment

As said above, the polar head is known to protrude out the binding groove of the CD1d and is a key element in the TCR recognition. Its orientation is characterized by three dihedral angles: φ_x_, φ_y_, φ_z_. Therefore the ability of the GAFF force field to reproduce static ab initio quantum chemistry calculations was tested on these three degrees of freedom. Focusing on these three parameters, rigid potential energy scans for internal rotations about the three axes φ_x_, φ_y_, φ_z_ in the 8 compounds were performed to compare GAFF energies to electronic structure calculations (at the DFT/6-31G* level of theory using the M06 hybrid functional, which is recommended by Truhlar and col. [39] for non-covalent bonds descriptions). Single points energy calculations were performed every 5°. It can be clearly seen from Figure S1 that the potential energy profiles for these three rotational degrees of freedom are reproduced in a satisfactory manner by the force field GAFF. The rotations around the two first angles (φ_x_, φ_y_) emphasize wide regions of steric hindrance (several tens or even several hundreds of kcal/mol) corresponding to steric clashes between the head group and the osidic bond, the sphingosine and acyl chains. Outside these very repulsive zones, GAFF correctly describes the minimum energy regions. Our tests were limited to these three internal coordinates. Indeed, it is to be noticed that in these binary complexes, the lipid anchors and the hydrogen bonds considerably reduce the other internal degrees of freedom of the ligand.

### 2D-RMSD, RMSF

A 2D-RMSD graph represents the root mean square deviation (RMSD) of every conformation to all other conformations of a simulation, as a function of time during a 240 ns simulation. Each point of the two-dimensional plot corresponds to the RMSD between two trajectory structures, and its value is encoded into a color. The diagonal elements (black) represent self-comparison (zero RMSD) and the yellow dots show the largest pairwise RMSD. RMSD values have been computed based on the binding region, i.e., using an atom selection including the alpha carbons of helices α_1_ and α_2_ only (residues in the range 58–92 and 137–184 in human and in the range 58–92 and 139–186 in mouse). The average of all RMSD values of this matrix was also computed for each graph. The resulting number represents the level of fluctuation of the whole system during the simulation. In parallel, the root mean square fluctuation (RMSF) of the CD1d protein was computed as a measure of the average atomic mobility. It was calculated on a residue basis using the RMSF of the positions of the C_α_ atoms of all the protein.

### Dihedral footprint, inter-helix distance

A so-called dihedral footprint was computed, based on 9 dihedral angles made by the C_α_ atoms of 9 CD1d residues that form a contact with TCR. The considered residues are: 76, 79, 80, 83, 84, 87, 99, 147(149 in mouse) and 150(152 in mouse). Sine and cosine transformed dihedral angles were used to avoid problems arising from the circularity of angles. The human 2PO6 X-ray structure was chosen as the reference in all simulations for the calculation of the dihedral values. For one given dihedral angle θ_i_, the squared deviation from its reference position θ_0_ is measured as follows: 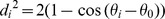. This formula derives from the sum of the two squared differences for sine and for cosine. The final index was obtained by calculating the root mean square deviation over all the 9 dihedral angles. This index varies in the range 0–2 and the percentage change is reported in the figures.

The change in inter-helix distance was monitored by computing the distances between centroid pairs along the two portal helices α_1_ and α_2_. Each centroid is formed by four successive residues. Helices α_1_ and α_2_ consist of 7 and 10 centroids, respectively. The chosen procedure is based on a modified Hausdorff distance calculation. For every centroid of helix α_1_ we determine the smallest distance to any centroid of helix α_2_. The sum of all these distances is computed. The procedure is repeated for every centroid of helix α_2_ relative to all points of helix α_1_ and a second sum is deduced. Finally, the inter-helix separation was assessed by summing these two distances and by averaging over the total number of centroids of helices α_1_ and α_2_.

### 1D_FEL, 3D_FEL

Each dihedral angle θ_i_ formed by 4 bonds joining 4 successive C_α_ atoms along the main chain of the protein CD1d is a probe of the free-energy landscape (FEL) along the primary sequence. The 1D Free Energy Landscape (1D_FEL) [40] of these coarse-grained dihedral angles are obtained based on the logarithmic relation at 300K: G = −RT ln P(θ_i_) using the one-dimensional probability distribution function P(θ_i_) derived from our simulations. For a given dihedral angle, in order to compare the 1D_FEL between two simulations i and j, the following procedure was carried out. First, the 1D_FEL curve j was shifted on the energy axis so as to align the minima of i and j. Next, the curve j was shifted on the angle axis so as to minimize the Hausdorff-like distance between the two curves i and j. In this way, the resulting distance value (with unit mixing the energy and angle axes) measures shape dissimilarity between the two 1D_FELs. Points with energy above 20 k_B_T in the 1D_FEL were excluded from this procedure.

Similarly, for the polar head, three-dimensional histograms were constructed from values of dihedrals φ_x_, φ_y_, φ_z_ and converted to free energies (3D_FEL) based on an analogous logarithmic relation at 300K using the probability distribution function P(φ_x_, φ_y_, φ_z_) given by our simulations. Each of the isosurfaces shown in our paper corresponds to points of the 3D-space (φ_x_, φ_y_, φ_z_) with a constant free energy isovalue (in kcal mol^−1^). Nine isovalues were considered from 1 to 9 k_B_T.

Our study disclosed the existence of specific states with lifetime ranging from a few nanoseconds to 30ns. It takes only a few picoseconds to switch from one of these states to the other. Due to this very short timescale, no dihedral principal component analysis (dPCA) could be carried out to correlate the occurrence of these transitions to specific structural and dynamical features of the system (coordinates of the system were written every 1 ps during the MD).

## Results and Discussion

### Detailed analysis presented on the systems human CD1d-KRN7000 and empty human CD1d

To achieve our objectives, a large conformational sampling is required. Such a large amount of data is difficult to analyze. Our trajectory analyses use a wide range of tools to extract the most relevant and unanticipated events. Since we are interested in detecting conformational behavior differences between systems, these tools are now exemplified in the case of the two systems **Sy1** and **Sy16**, expected to behave quite differently.

**Figure 3:**
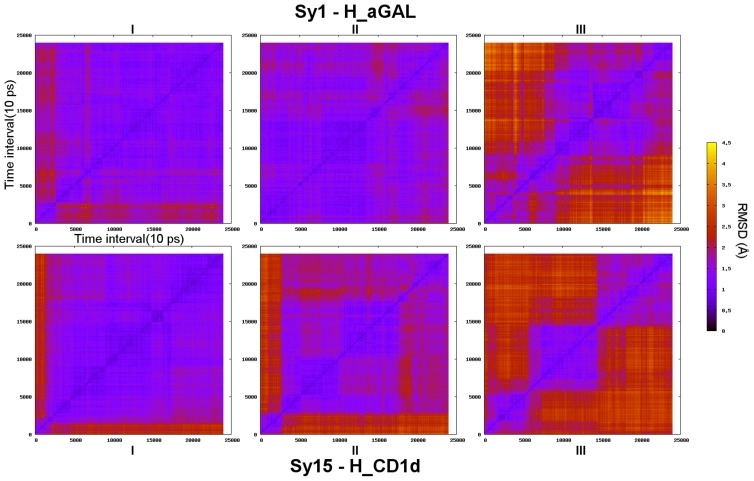
Root mean square deviation matrix of H_aGAL (top) and H_CD1d (bottom) simulations. Root mean square deviation (alpha carbons of CD1d α_1_/α_2_ helices) of every conformation to all other as a function of time during a 240 ns simulation; replica I, II, III.

First, 2D-RMSD (α_1_/α_2_ interface) can be helpful to detect different conformational “substates”. While replicas I and II of H_aGAL show a relative homogenous 2D-RMSD plot (Figure 3), replica III appears to move into two “wide” different conformations. Trajectories of H_CD1d give more contrasted patterns, with replica III showing three dissimilar clusters. The matrix average of RMSD values accurately reflects the level of granularity observed by visual inspection (numbers reported in Figure S2). These plots clearly show that the two CD1d helices can be very stable during 240 ns or in contrast go through several distinct conformational states with a life of a few tens of nanoseconds. Therefore, it seems that one cannot just perform a single 240 ns MD simulation but rather running multiple MD with different starting initial conditions is better to study the interface of the binary complex. Surprisingly, at this stage, though H_CD1d displays slightly more contrasted 2D-RMSD figures, the α_1_/α_2_ interface of H_aGAL and H_CD1d does not appear to have greatly differing dynamical behavior.

**Figure 4:**
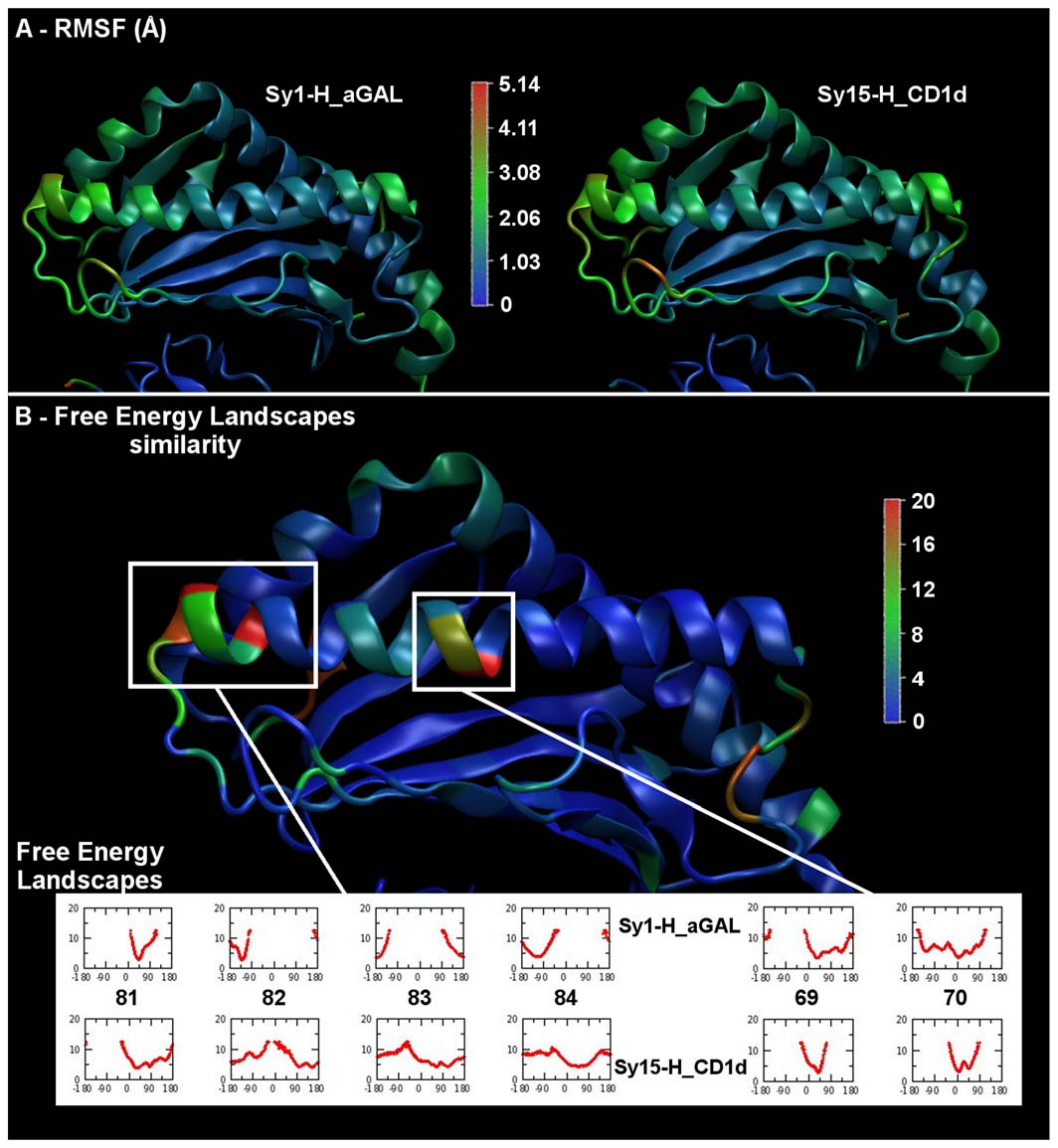
Root mean square fluctuation and 1D-FEL dissimilarity of H_aGAL and H_CD1d systems. (A) per residue RMSF (Å) of binary complexes H_aGAL and H_CD1d for replica III; color scale (from blue to red) is proportional to the fluctuation (B) 1D-FEL dissimilarity for each coarse-grained dihedral angle along the main chain between binary complexes H_aGAL and H_CD1d (replica III); color scale (from blue to red) is proportional to the dissimilarity measured by a Hausdorff-like distance (unit mixing the energy and angle axes); the free energy profiles of the six residues the most influenced by the presence of **1** are emphasized below the protein; units are k_B_T (energy) and degrees (angles).

Despite the presence of ligand **1** in the case of H_aGAL, a comparison of the per-residue RMSF calculated from the binary complex trajectories shows that the two systems (with or without ligand) undergo very similar fluctuations (Figure 4, panel A), in particular no appreciable difference is observed in the ligand-binding pocket. For both systems the linker domain (amino acid sequences separating the helices domain from the beta sheet part of the CD1d protein) is the most flexible region, as expected. The extremities of the helices show the highest mobility, beta sheets are very stable. The same conclusions arise for the three replicas. The advantage of this tool over 2D-RMSD is to localize the regions of high mobility. However, it does not permit to distinguish between two situations: (a) regular but larger fluctuations due to higher mobility of some residues at the local scale in specific region of the interface but maintaining the secondary structure or (b) direct loss of secondary structure elements occurring at one point of the simulation, involving a larger amplitude motion and impacting RMSF values.

The analysis of the 1D Free Energy Landscape (1D-FEL) of the Coarse-Grained Dihedral Angles of the protein (CGDA, defined by four successive alpha carbon atoms) was used with a view to detecting the presence of such possible structure deformation. This methodology has been recently applied to studies of proteins. [40] In order to capture the largest deformations in the protein resulting from the absence of ligand we decided to compute a Hausdorff-like distance (see Materials and methods section) that measures how far two Free Energy Profiles (FEP) are from each other for a given dihedral angle in H_aGAL or in H_CD1d. The deformations can be well appreciated in panel B of Figure 4 where the color of the ribbons is directly proportional to this distance acting as a dissimilarity index. We report at the bottom of Figure 4 the FEP of the six residues the most influenced by the presence of **1**. As can be seen, significant anharmonicity appears at the end of the helix α_1_ (on the side towards the F′ pocket) when the ligand is lacking. This comes in conjunction with the middle of the helix being more rigid. The lack of ligand clearly produces a bend in the middle of helix α_1_ combined with the destructuration of its extremity.

**Figure 5:**
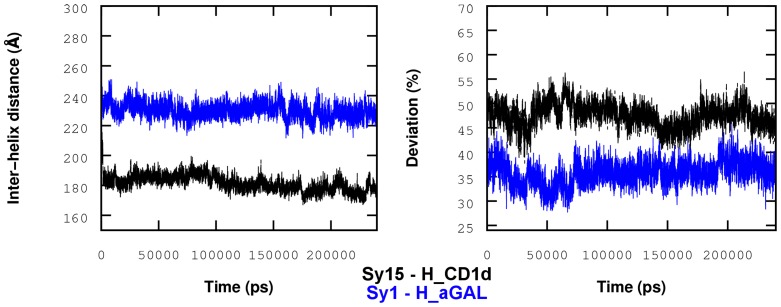
Inter-helix distance and dihedral deviation index. Left: inter-helix distance measured as the distance between centroids of α_1_ and α_2_ helices; Right: deviation from the interface X-ray structure based on TCR-CD1d contacts dihedrals; lipid-free CD1d (black) and **1** loaded CD1d (blue); replica II.

One may now question how this deformation will affect the binding footprint between the TCR and CD1d. The evolution of this interface during the simulation was monitored according to two complementary indexes calculated with reference to the X-ray structure (PDB 2PO6). In Figure 5, the first index (the distance between the centroids of α_1_ and α_2_ helices) reveals the closure of the cavity entrance in the lipid-free system (H_CD1d). Secondly, a so-called dihedral footprint was computed, based on the dihedral angles made by the successive alpha carbons of the 9 CD1d residues that are known to form a contact with TCR. This index varies in the range 0–2 and the percentage change is also reported Figure 5 (right). According to this index, the ligand-free CD1d interface greatly deviates from the reference (X-ray structure), by about 50% in comparison with the H_aGAL system (35%). All these results are in agreement with the theoretical results of Garzón et al. [41] who observed the same trend.

**Figure 6:**
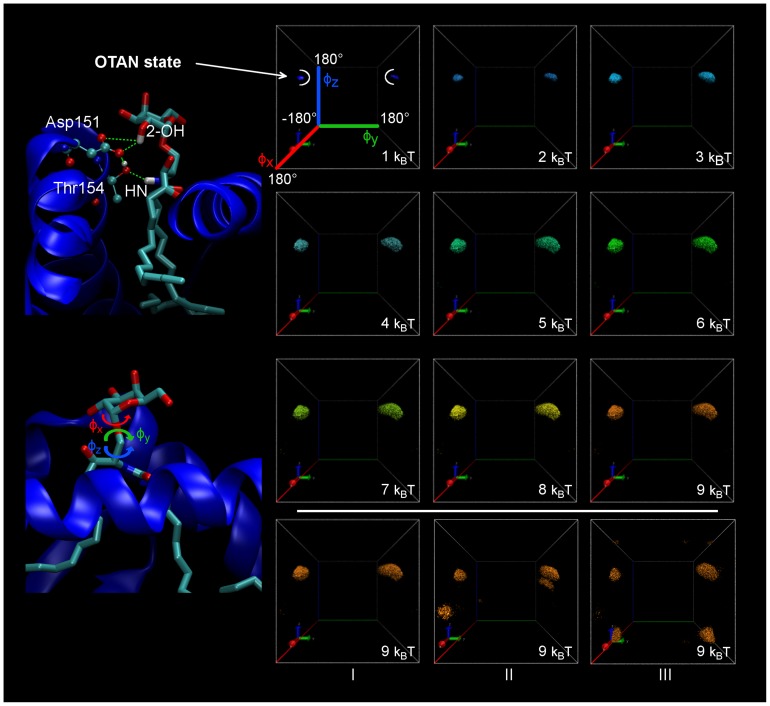
3D-FEL associated to the polar head conformations for the H_aGAL system. Left: **1** loaded human CD1d with the OTAN hydrogen bond network emphasized and the three torsion angles for the polar head to be rotated. Right: 3D-free energy representations of H_aGAL system **1** (replica I, T = 300K); each of the nine isosurfaces corresponds to points of the 3D-space (φ_x_, φ_y_, φ_z_) with a constant free energy isovalue (in kcal.mol^−1^); the three last boxes correspond to the three isosurfaces (2.7 kcal.mol^−1^) obtained for the three replicas (240 ns each).

Finally, the conformational space explored by the polar head of the ligand during the simulations of H_aGAL was described using the three torsion angles of the three successive rotatable bonds shown in Figure 6. In the following, the first one (φ_x_) will be referred as the anomeric pivot, the second one (φ_y_) as middle pivot and the last one (φ_z_) will be denoted the amide axis. Theoretically, rotations around φ_x_ and φ_z_ yield conformations in which the polar head has rotated horizontally at the top of the cavity entrance. In the CD1d environment, these two motions should be hardly sterically hindered by the two helices. In contrast, rocking about the middle pivot will get the polar head striking the helices. In all cases, such individual rotations would require the loss of the OTAN H-bond network. The free energy landscape of the MD trajectory along these three coordinates is illustrated in Figure 6 for the system **Sy1**. The top panel shows all points in the (φ_x_, φ_y_, φ_z_) space with isovalues of G being one to nine k_B_T (0.6 to 5.3 kcal.mol^−1^) for replica I of H_aGAL. For all simulations, the conformational space does not grow any more for energies above 9 k_B_T. For replica I, the 3D-FEL emphasizes only one conformational state, showing that the OTAN network is strong enough to maintain the orientation during all the 240ns simulation. This state, hereafter referred to as “OTAN state”, is centered about the φ_x_, φ_y_, φ_z_ coordinates: (50°, 157°, 51°) in replica I. Comparison with the three replicas of H_aGAL at G = 5.3 kcal.mol^−1^ (9 k_B_T, bottom panel of Figure 5) reveals the presence of the OTAN state in every case. But in addition, two secondary conformational states can be visited, mainly limited to the (φ_x_, φ_z_) plane. It is very important to note that, as with the OTAN state, these two complementary states maintain the polar head interacting with helix α_2_. Actually, the second state observed in replica II is a combination of two rotations about φ_x_ and φ_z_ that limits the displacement of the sugar ring (see Figure S3). The third state observed in replica III corresponds to a rotation of about 180° around φ_z_, which brings again the polar head in contact with helix α_2_ (see Figure S4). In this last state, the Asp151 residue is now hydrogen-bonded to the 4′-OH group (sphingosine chain) and the Trp153 side chain is again in van der Waals (VDW) contact with the hydrophobic side of the polar head. But most importantly, no conformations are observed involving rotations about the middle pivot φ_y_, points that would lie inside the 3D-FEL box, involving the “y” axis.

### Human versus mouse

Considerable structural similarities are apparent between mouse and human CD1d molecules. Focusing on the α_1_ and α_2_ helices-binding domain, human and mouse amino acid sequences show a similarity of 81.2% and an identity rate of 65.2% in this portion of the protein. The amino acid sequence is highly conserved in particular the residues Asp80, Asp151(153 in mouse) and Thr154(156 in mouse), which are of crucial importance to bind the ligand through the OTAN network, are present in both proteins. There are 25 amino-acid variations located in the TCR-interface domain. One of these seems to be particularly important. At position 153, the crystal structures show the presence of a bulky tryptophan side chain in human CD1d (Figure 1) in contrast with the glycine (no side chain) in mouse CD1d. This Trp153 side chain is in VDW contact with the hydrophobic side of the sugar in a face-to-face configuration (see Movie S1) and one may question whether the absence of this residue in the mouse CD1d may exert an indirect effect on TCR binding or not. Actually, as can be seen in Figure 1, the galactose head group acts as a mechanical stop restricting the TCR approach to only one side: the F′ part of the CD1d groove.

Both human and mouse CD1d proteins are able to induce a balanced Th1/Th2 response depending on the loaded ligand. However, the biological activities of analogues of **1** loaded into mouse or human CD1d can be sometimes quite different. [14], [32]

**Figure 7:**
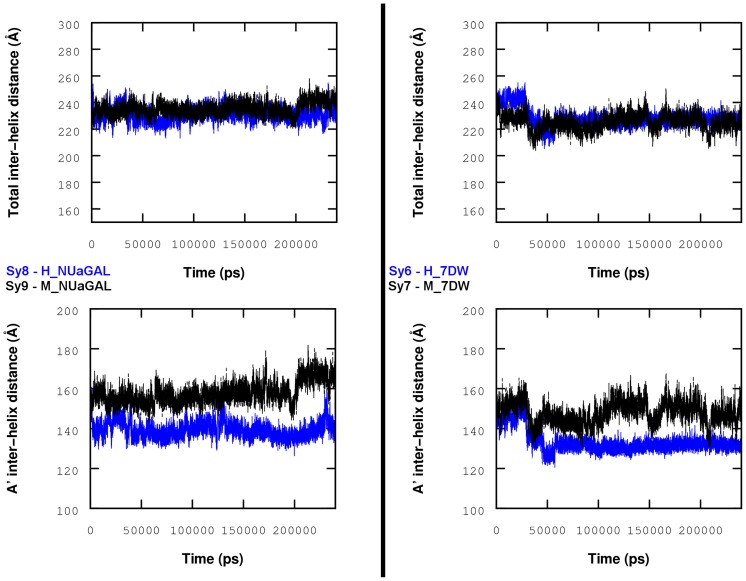
CD1d inter-helix distances for H_NUaGAL, M_NUaGAL, H_7DW and M_7DW systems. This distance is measured as the distance between centroids of α_1_ and α_2_ helices; Replica I.

Hence, four “human versus mouse” comparisons are conducted for systems: **Sy1**/**Sy2**, **Sy3**/**Sy4**, **Sy6**/**Sy7** and **Sy8**/**Sy9**. These systems were chosen because, with ligand unchanged, human and mouse CD1ds produce the same polarization (Th1 or Th2). Moreover, to ensure a thorough comparison, the ligand **2** is supplemented in both human and mouse simulations with a “spacer”, i.e. a linear hydrophobic compound taken from the CD1d-**2** PDB (3ARB). From the analyses of these 24 trajectories, the outstanding results are the following. From a structural point of view, in all CD1d mouse simulations, we clearly observe an increased inter-helix distance localized on the A′ pocket side. On average, the inter-helix distance is about 1.3 Å larger in mouse simulations than in human CD1d simulations (see Table S1). This is not surprising since this portion of the two helices concentrates ten amino acid variations (8 residues on α_2_ helix and 2 on α_1_ helix) involving residues with physico-chemical properties very different between human and mouse proteins. This effect is however counterbalanced on the F′ pocket side where the portions of helices α_1_ and α_2_ come closer such that overall, the total inter-helix distance is almost the same for human and mouse CD1ds during the simulations (see Figure 7 and Table S1). Curiously, the M_NUaGAL/H_NUaGAL comparison exhibits a still larger increase of the inter-helix distance: 2.1 Å larger in mouse simulations than in human. This is likely to be correlated with the fact that the naphthyl group introduced on the head part of this analogue facilitates the interaction with two residues in a small pocket between helices α_1_ and α_2_ of CD1d (as demonstrated by Trappeniers et al. [30]). One of these residues is Ile69 in humans, replaced by Me69 in mice. Finally, it is to be noted that this inter-helix variation observed between mouse and human CD1ds is not accompanied by a dihedral footprint deviation (measured as a percentage value from our analysis tool based on CD1d contacts).

From a dynamical point of view, a higher mobility is found for the human protein CD1d compared to mouse CD1d. This is slightly noticeable from the 2D-RMSD (see Figure S2) and from RMSF pictures where more fluctuations are observed for the human linkers. From the 3D-FEL analysis tool, comparing the motions of the polar head in mouse or human CD1d, all simulations emphasize more flexibility when the ligand is loaded into the human CD1d rather than in the mouse CD1d. Whether for human or mouse, there is predominantly only one conformational free energy well explored during the simulations, which we term the “OTAN state” (Figure 6). However, the conformational space sampled within the mouse simulations is significantly smaller than the one for human simulations. This is evidenced by estimating the corresponding volumes enclosed by the free energy isosurface (9 k_B_T) in the 3D representation (φ_x_, φ_y_, φ_z_) for both mouse and human simulations (see Table S2).

Unexpectedly, we observe a very intriguing free energy landscape for the replica II simulation of human **2**, indicating an ensemble of five conformational states. This will be discussed in more details in the next section.

### Th1 versus Th2

The goal is now to find out correlation at the molecular level, if any, between chemical modulation of the ligand and the orientation of the known biological response, Th1 or Th2. Analogue **1** (Th1) is compared to **2** (Th2), **7** (Th2) and **8** (Th2) within three comparisons. Firstly, two comparisons are made with the ligand loaded into the human CD1d: (a) H_aGAL/H_OCH or (b) H_aGAL/H_SaGAL. Additionally, in mouse CD1d, the compared behavior (c) M_aGAL/M_AZOL is then addressed. This study focuses on the systems **Sy1**, **Sy2**, **Sy3**, **Sy12** and **Sy14** of Table 1.

**Figure 8:**
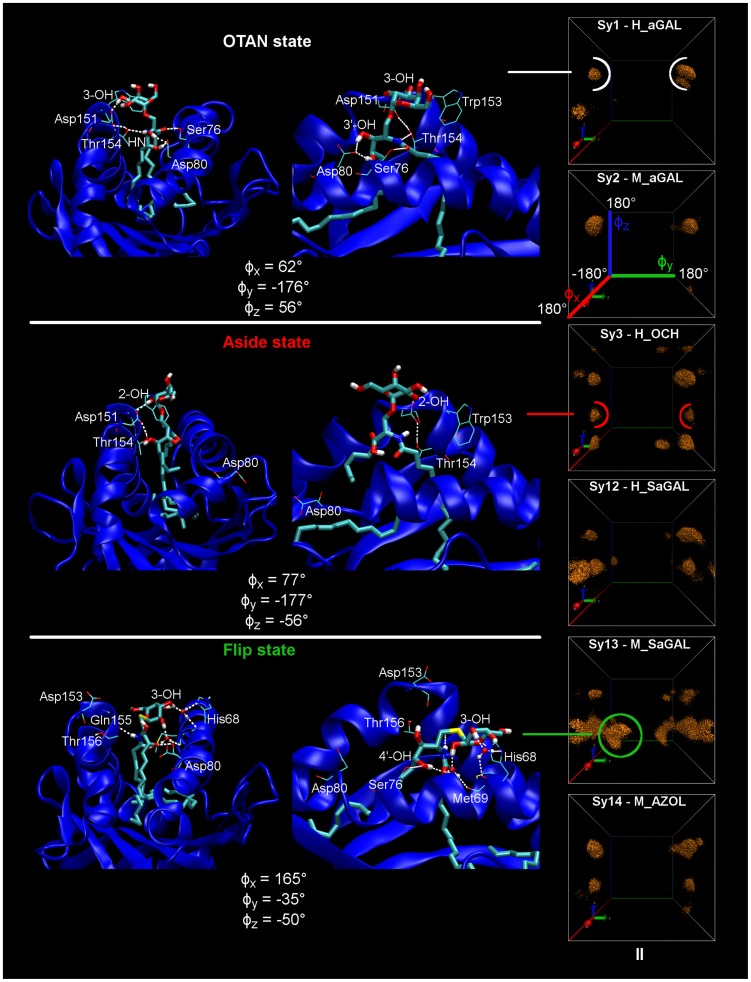
3D-free energy representations of systems Sy1, Sy2, Sy3, Sy12, Sy13 and Sy14. Each isosurface corresponds to points of the 3D-space (φ_x_, φ_y_, φ_z_) with a constant free energy isovalue of 5.4 kcal.mol^−1^ (9 k_B_T); replica II (240 ns) at T = 300K.

The key results are the following. From a structural point of view, H_OCH and M_AZOL, simulations revealed significant structural changes of the CD1d protein with regard to the other simulations. The replica II (mainly) of the H_OCH simulation shows a modification of the α_1_ helical structure involving residues 74 to 82 (on the F′ pocket side) as revealed and evidenced by the 1D-FEL analysis (it can be seen on Figure 8, middle panel). Overall, human CD1d in complex with **2** appears to be slightly more fluctuating compared to H_aGAL simulations. More specifically, the residues on the F′ pocket side display a higher mobility in the RMSF analysis of H_OCH. All these changes are very likely due to the truncated sphingosin chain of **2**, even though a spacer lipid complements the F′ pocket in this case. The presence of the ligand **8** in the human CD1d protein causes a pronounced enlargement of the 1D-FEL for dihedrals around the residue 153 (residues 151 to 155 of helix α_2_). This was strongly observed for all three replicas of the M_AZOL system, compared to the M_aGAL one. This clearly shows that the replacement of an amide function with a triazole group significantly increases the flexibility of the α_2_ helix and consequently disturbs the OTAN hydrogen bond network, which involves the residues Asp151 and Thr154 (helix α_2_, human numbering). A polar head destabilization is then expected (discussed hereafter). Concerning the ligand **7**, no CD1d structural change has been observed here. In spite of the two aforementioned structural changes, absolutely no CD1d difference has been observed concerning the binding footprint and the inter-helix distance between all these systems, which could have explained a Th2 bias.

We turn now to the discussion about the dynamical features of the polar head. Very interestingly, the three ligands (**2**, **7**, **8**), which are known to induce a Th2 bias, display multiple well 3D-FEL (**Sy3**, **Sy12**, **Sy13** and **Sy14** on Figure 8) compared with ligand **1** (Th1 bias in human and mouse CD1d, **Sy1** and **Sy2** on Figure 8). H_OCH simulations exhibit this feature only once, for replica II. This suggests that at least three trajectories or more are needed to reveal and confirm such a conformational behavior. A common pattern to these three ligands is a new conformational state, which appears about 1.8 kcal.mol^−1^ above the OTAN state, designated as the “aside” state in Figure 8. The polar head has rotated by about 110° relative to the φ_z_ axis in the resulting conformations. In these conformations, the OTAN network and the hydrogen bond with Asp80 are lost, or partially lost. The polar head points now toward the α_1_ helix but no direct contact has been observed between the sugar and this helix. In fact, the two residues Val72 and His68 of helix α_1_, which face the polar head, are positioned too far away to attract the ligand. In other words, the source of the new observed state is not helix α_1_. Thus, it seems that a chemical alteration is able to make accessible the “aside” state, thanks to the CD1d interaction.

It should be noticed that the VDW contact initially made by the hydrophobic side of the sugar with Trp153 in the OTAN state (Figure 1), is also lost in the “aside” state. The new polar head orientation clearly penalizes a TCR recognition process. But this reorientation towards helix α_1_ is found to be reversible. The lifetime of this state ranges from a few nanoseconds to 30 ns. It takes only a few picoseconds to change from the OTAN state to the “aside” state and vice versa. It is difficult to correlate the occurrence of this transition to specific structural and dynamical features at such a short scale, but obviously, the new state arises in conjunction with the partial or total loss of the OTAN network and the lost of hydrogen-bond between 3′-OH and Asp80. Also, rare and slight rotations around the middle pivot φ_y_ can occur. From an energetic point of view, the OTAN state remains the ground state in all our simulations (except in the case of M_SaGAL that will be discussed below). For the H_SaGAL simulations, the “aside” state is also present. But moreover, the free energy landscape shows unstructured features above 2 kcal.mol^−1^, indicating that all conformations are frequently visited along the “x” axis in a broad basin (Figure 8). In other words, in these high-energy states, the polar head is almost free to move around φ_x_.

Extending this 3D-FEL analysis of the polar head dynamics to all studied systems leads to very interesting results. All the binary systems known to have a Th1 biased response (**Sy1**, **Sy2**, **Sy6**, **Sy7**, **Sy8**, **Sy9**) never show the “aside” state, nor such multiple-well energy landscapes in the (φ_x_, φ_y_, φ_z_) space, for any replica. In contrast, all the other seven loaded CD1d systems (but **Sy4** and **Sy10**) for which a Th2 biased polarization has been demonstrated, are characterized (at least once) by a multiple-well landscape including the specific state above-mentioned. This concerns the systems **Sy3**, **Sy5**, **Sy11**, **Sy12** and **Sy14** in Table 1. One may question whether it is a pure coincidence. Obviously, despite our very large-scale MD simulations, a more representative statistical sample of MD trajectories is required to further reinforce this trend (typically, more than three replicas would be needed). But, our results are consistent with the accepted model that correlates a Th2 biased response to chemical modulations yielding a less stable CD1d-glycolipid complex. More precisely, we show here that the complex instability results from increased sugar head fluctuations above the CD1d binding groove that will hinder TCR recognition. All the simulations showing an “aside” state are associated with a Th2 response exclusively.

More interesting is the simulation of the M_SaGAL system, which displays a very particular 3D-FEL illustrated at the bottom of Figure 8. All three replicas also reveal multiple energy wells with broad basins and the so-called “aside” specific state is present. But it is the only system for which the OTAN state is not the ground state. Actually, the specific “aside” state is observed to be the lowest one in the 3D-FEL analysis of M_SaGAL, the OTAN state being about 1.2 kcal.mol^−1^ above it. Furthermore, in our study, it is the only system that displays conformations involving large rotations of the polar head around the middle pivot φ_y_, resulting in points distinctly inside the 3D-FEL box. A typical conformation is illustrated in Figure 8. It is associated to a third distinctive state we call the “flip” state in which the polar head hydrogen bonds to the α_1_ helix. Let us recall that rotations about this φ_y_ axis would normally drive the polar head in contact with the helices. Such motions are clearly hindered in all other studied systems. This means that in this case, the polar head of the thio-galactoside derivative slightly pulls out of the CD1d host during the M_SaGAL simulation. Relative to the C–O, the C–S bond is longer. Combined with a greater separation inter-helix distance observed on the A′ pocket side of mouse CD1d, this is probably the reason why we observe this specific dynamical behavior for the M_SaGal simulation. Obviously, these slightly “extricated” conformations will prevent the TCR binding. This theoretical result fully agrees with biological evaluations [23], [31], [32] of this compound that show no activity against mouse iNKT cells.

In this general analysis, it is to be noted that, although they are known to give a Th2 biased response, the two binary systems **Sy4** (M_OCH) and **Sy10** (H_GOF) do not display the “aside” state. Additional replicas might be necessary to observe such behavior. However, in our simulations, these two systems with a truncated glycolipid chain (acyl or sphingosin) involve a strong change in the 1D-FEL of dihedrals on the helix α_1_ bearing the contacts, which are critical for CD1d-TCR recognition. Clearly, anharmonic flat or double-wells appear in the 1D-FEL of these dihedral angles. This suggests that under chemical modulation not only the polar head flexibility but also the deformation of helix α_1_ itself can deteriorate the TCR recognition process.

### Influence of spacer lipids and of the presence of the polar head

When short chain lipids (in analogues) are bound to the protein, spacer lipids may be simultaneously present in the pockets of CD1d. For example, two X-ray structures (PDB references 3ARB and 1Z5L) show the presence of A′ and F′ pockets spacer lipids when CD1d is partially occupied by the sphingosin truncated **2** and the acyl truncated **6** analogues, respectively. In the absence of the glycolipid ligand, endogenous ligands can also fill the entire volume in order to maintain stability and prevent protein denaturation [28]. It was then interesting to examine how the presence or the lack of spacer lipid impacts the polar-head dynamics and the conformations of the CD1d surface.

Firstly, the influence of the presence of a spacer is addressed by comparing the trajectories of the systems **Sy3** (with **2**+ lipid) and **Sy5** (with **3**). The two ligands are almost identical, the molecule **3** having only two CH_2_ more in its acyl chain. The tremendous difference is that in the system **Sy3**, an additional linear hydrophobic compound C_12_H_26_ is present in the F′ pocket in addition to **2**. As expected, 2D-RMSD and RMSF analyzing tools indicate a higher fluctuation for the system lacking the spacer lipid. This increased flexibility concerns the CD1d residues in the vicinity of the F′ pocket. Also, in the absence of spacer lipid (**Sy5**) the inter-helix distance becomes slightly larger (by about 0.3 Å, see Table S1). In addition, the absence of a spacer lipid causes a slightly larger conformational space of the polar head in the 3D representation (φ_x_, φ_y_, φ_z_) of **3** compared to **2** (see Table S2). Both systems reveal the specific “aside” state on their 3D-FEL figure, but the simulation without complementary free lipid displays broad basins with the polar head almost free to move around the φ_x_ axis (see H_OCH and H_OCH9 3D-FELs in Figure S5). All these results are consistent with the experimental findings of Garcia et al. [42] who concluded that the spacer lipid appears to work in concert with the ligand to stabilize the binding groove.

How the presence of the polar head impacts on the molecular dynamics was addressed based on the comparison between simulations **Sy1** and **Sy16** (human CD1d with two headless lipids filling simultaneously pockets A′ and F′). As expected, the observation of RMSF figures shows that fluctuations are more important in the middle of helix α_2_ when the polar head is missing. But further, fluctuations also tend to affect helix α_1_ in this case. This shows the importance of the presence of the OTAN hydrogen bond network for stabilizing the binding groove and then the CD1d surface in view of recognition by TCR.

### Conclusions

From the analysis of 48 trajectories, it appears that the α_1_/α_2_ inter-helix distance differs in mouse and human loaded-CD1d simulations. A greater separation distance is observed on the A′ pocket side of mouse CD1d where strong residue dissimilarities appears between human and mouse helices. This point may be interesting in view of the known differences in cytokine profile production that sometimes appear between human and mouse systems. For the lipid-free CD1d simulations, we observe the spontaneous closure of the binding domain entrance, accordingly with previous theoretical results. [41] In complement, a spacer lipid simultaneously present with the ligand stabilizes the F′ pocket. In a similar manner, we show the key role of the polar head in stabilizing the binding groove at the CD1d surface.

The goal of this study was to get insight into the impact of a chemical variation of molecule **1** on the structure of the binary complex formed between the ligand and protein CD1d. A crucial point was how this could affect the CD1d binding footprint with possible deterioration of the TCR recognition. The major result of our study is that the dynamical behavior of the polar head seems to be a key factor when trying to correlate a ligand modulation with the orientation of the known biased biological response, Th1 or Th2. Considering three successive dihedral degrees of freedom (φ_x_, φ_y_, φ_z_), which govern the polar head rotation above the CD1d binding groove, our simulations permitted to identify three model situations. In the first one, the conformations visited by the polar head during the simulation mainly fall in a portion of the free energy landscape we call the “OTAN” state. It corresponds to the polar head hydrogen-bonded to the CD1d through the well-known H-bond network built up from 2-**O**H, **T**hr154, **A**sp151, and amide-**N**H. Only a few higher free energy states can appear, but all of these maintain the polar head in contact with helix α_2_. Sampled conformations are exclusively restricted to well-separated (φ_x_, φ_z_) minima corresponding to rather structured states. A second situation corresponds to the emergence of a specific conformational state in the energy subspace (φ_x_, φ_z_), about 1–2 kcal.mol^−1^ above the OTAN state. This new “aside” state is characterized by the polar head pointing toward the α_1_ helix, but without direct interaction with CD1d. It arises in conjunction with the partial or total loss of the OTAN network and the loss of the hydrogen bond between 3′-OH and Asp80. The new polar head orientation clearly penalizes the presentation to TCR. The lifetime of this state ranges from a few nanoseconds to 30 ns. The ligands associated with Th1 biased response never displayed this “aside” state in their free energy landscape in any simulation replica. By contrast, all the simulations showing an “aside” state are associated with a Th2 response exclusively. Obviously, the previous 10 ns simulations of Henon et al. [17] were missing this conformational space. Sampling has been improved here with longer trajectories but also mainly by running several independent simulations, thus exploiting different starting conditions. Three 240 ns trajectories have been produced for each of the 16 systems (0.72 µs each). Only on this time scale and using multiple replicas could the emergence of these specific states be disclosed. But a still more representative statistical sample of MD trajectories would be required to further reinforce our findings. The interesting thing is that our model holds for very different chemical modulations affecting the anomeric bond as well as the polar linker, or either the sphingosin lipid chain. A third model situation arises when the specific “aside” state become the ground state, below the OTAN state. Then, at higher energies, new conformations appear in which the polar head turns upside-down and hydrogen bonds to the helix α_1_, a situation incompatible with TCR recognition. We observe then broad basins in the 3D free energy landscape of the polar head that must necessarily involve the slight extrication of the sugar head from the cavity entrance, correlated with no biological activity. In this case (thio-analogue of **1**), our findings indicate that mouse simulations behave differently than human ones.

The existence of a high-energy but populated “aside” state in the simulations of some analogues of **1** very likely contributes to reduce the stability of the ligand-CD1d binary complex. Binding free energy calculations using molecular dynamics tools could have potentially provide information. However, we presume that the very large number of degrees of freedom in these glycolipids would have prevented obtaining accurate results. Moreover a change in the binary complex stability does not systematically affect the affinity to TCR. Therefore, such binding affinity calulations are certainly not the best way to find out relationships here. The route we have chosen allowed unambiguous identification of these states.

Of course, it would be naive to believe that a molecular model can capture such a complex biological response. The mechanisms by which analogues govern the cytokine profile are multifactorial. For example, Sullivan et al. [43] showed that a critical parameter for a glycolipid to influence the cytokine response is its stability in cells. Overall, our results are consistent with the often-invoked model that correlates a Th2 biased response to chemical modulations yielding a less stable CD1d-glycolipid complex and hence a less stable ternary complex. Additionally, the 1D free energy landscape analysis tool permitted to show that not only the polar head but also modifications of α_1_ and α_2_ helical structures could result from chemical variations of molecule **1**.

Even though the chemical alterations seem large and significant, the set of molecules studied here have common structural features, what could limit the application of our methodology to other class of analogues. First, chemical alterations that prevent defining the three dihedral angles φ_x_, φ_y_, φ_z_ cannot be studied within our procedure. This concerns analogues that derive from specific osidic link variations. Moreover, it is clear that our results here only apply to analogues having a galactose residue. Another sugar group (glucose, …) would likely change the free-energy profiles in a way that cannot be predicted by our results obtained with the galactose component. Furthermore, our methodology only allows checking that preconditions for interaction of the binary complex with the TCR exists. From our study, these requirements for an efficient association are: a polar head dynamics with significantly populated OTAN state, and a limited deformation of helices α_1_ and α_2_. But our model (based on the CD1d-ligand system) cannot explicitly handle the interactions between TCR and the binary complex.

However, our polar head 3D-FEL tool combined with the 1D-FEL analysis of CD1d dihedrals in the binary complex provides a structural basis for predicting the very different dynamical behaviors of α-glycosphingolipids in CD1d and might aid in the future design of new analogues of **1**.

## Supporting Information

Figure S1 **Torsion energy profiles for the polar head of the 8 simulated ligands.** The rigid potential energy scans for internal rotations about the three axes φ_x_, φ_y_, φ_z_ were performed to compare GAFF force field energies to electronic structure calculations (DFT/6-31G* using the M06 hybrid functional).
(DOCX)

Figure S2 **2D-RMSD graphs for all the 16 simulated systems.** The average of all RMSD values of this matrix was also computed for each graph. The resulting number represents the level of fluctuation of the whole system during the simulation.
(DOCX)

Figure S3 **H_aGAL first secondary state.** This is a snapshot taken from the simulation of the H_aGAL system (replica I). The conformation of the polar head corresponds to a secondary conformational state different from the main OTAN conformation. But, as can be seen, the polar head is still attached to helix α_2_. Actually, the combination of the two rotations about φ_x_ and φ_z_ brings again the polar head in hydrogen contact with residues to helix α_2_. This state is not specific to the Th1 biological response and can appear in 3D-FEL of Th2 systems such as H_OCH9 or M_AZOL.
(TIFF)

Figure S4 **H_aGAL second secondary state.** This is a snapshot taken from the simulation of the H_aGAL system (replica III). The conformation of the polar head corresponds to a third conformational state. As can be seen, the polar head is still in contact with helix α_2_ (VDW contact with Trp153). This state showing a major rotation about φ_z_ axis is not specific to the Th1 biological response and can appear in 3D-FEL of Th2 systems such as H_OCH or H_OCH9.
(TIFF)

Figure S5 **3D-Free Energy Landscape of all systems at 9 k_B_T.** The conformational space explored by the polar head of the ligand during the simulations was described using the three torsion angles of the three successive rotatable bonds (starting from the anomeric bond). The resulting 3D Free Energy Landscapes are reported below.
(DOCX)

Table S1 **Inter-helix distance α_1_- α_2_ calculated either over 17 centroids, or over 11 centroids (on the A′ side of the CD1d protein in this case).**
(XLSX)

Table S2 **The volume enclosed by the free energy isosurface at 9 k_B_T in the 3D_representation (**φ**_x_,** φ**_y_,** φ**_z_) of all 14 loaded-CD1d systems.**
(XLSX)

Movie S1 **Molecular dynamics simulation (2 ns) of α-Galcer in H_CD1d.** This animation was built from the molecular dynamics simulation (sampled every 10 ps) of α-Galcer in human CD1d; it illustrates the van der Waals interaction of residue Trp153 (van der Waals representation) with the hydrophobic part of the sugar head; for the sake of clarity, H atoms and water molecules are not displayed.
(GIF)
