# Supplementary figures and images for: Relationships between Th1 or Th2 iNKT Cell Activity and Structures of CD1d-Antigen Complexes: Meta-analysis of CD1d-Glycolipids Dynamics Simulations
Source: PLoS Comput Biol. 2014 Nov 6;10(11):e1003902. doi: 10.1371/journal.pcbi.1003902 (PMC4222593; doi:10.1371/journal.pcbi.1003902)

**α-Galcer**


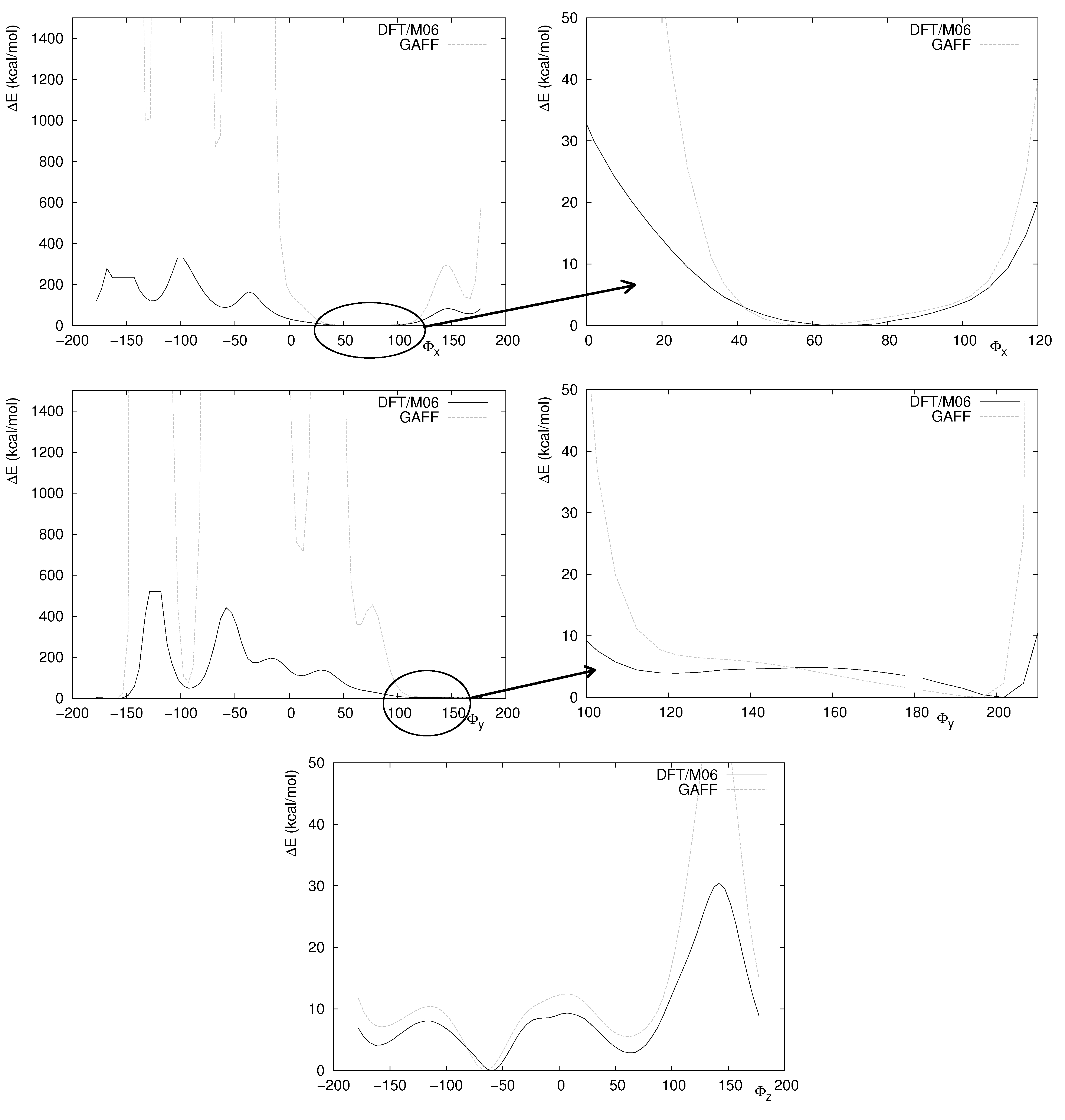


**OCH**


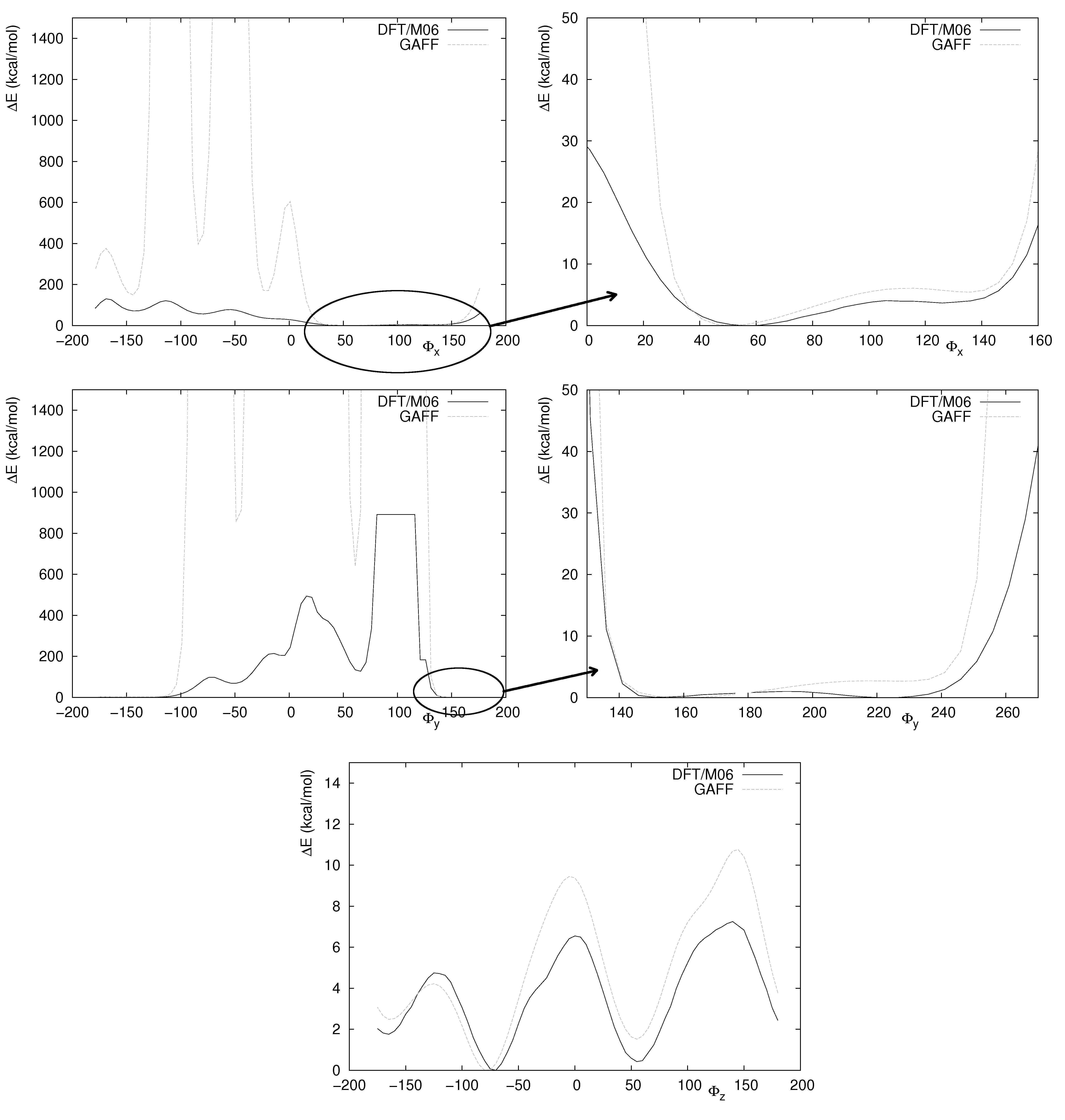


**OCH9**


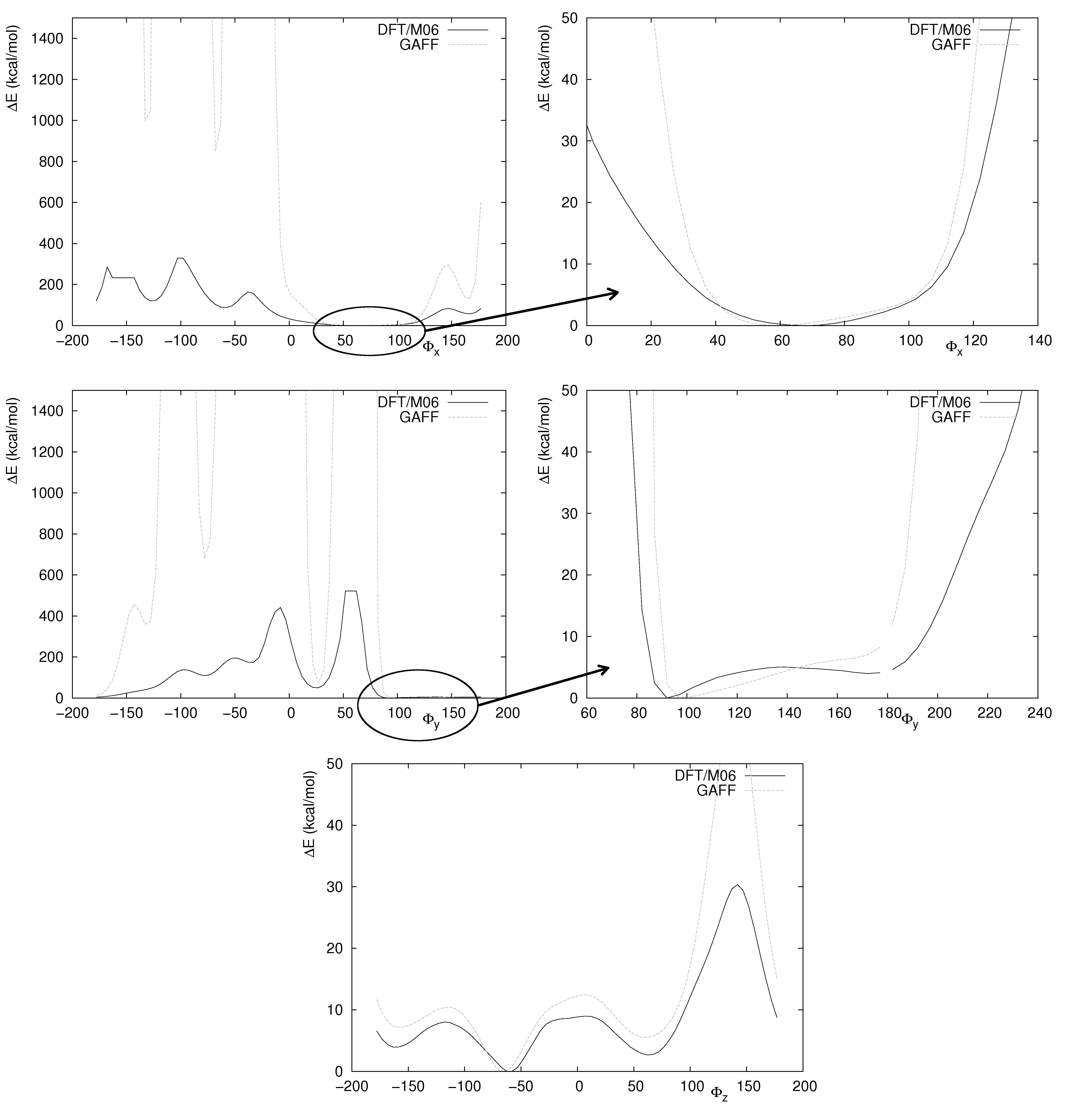


**7DW8-5**


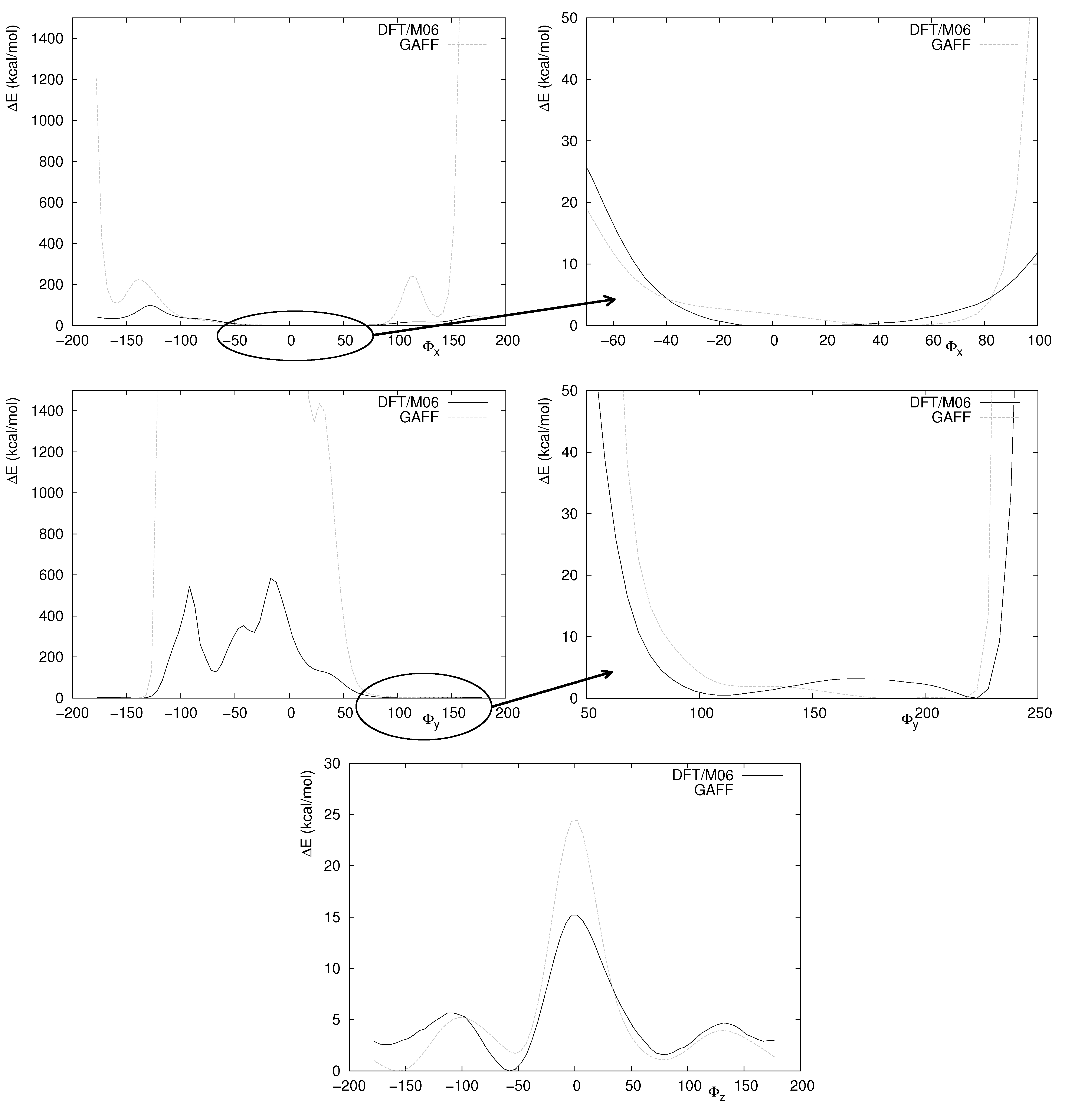


**Nu-α-Galcer**


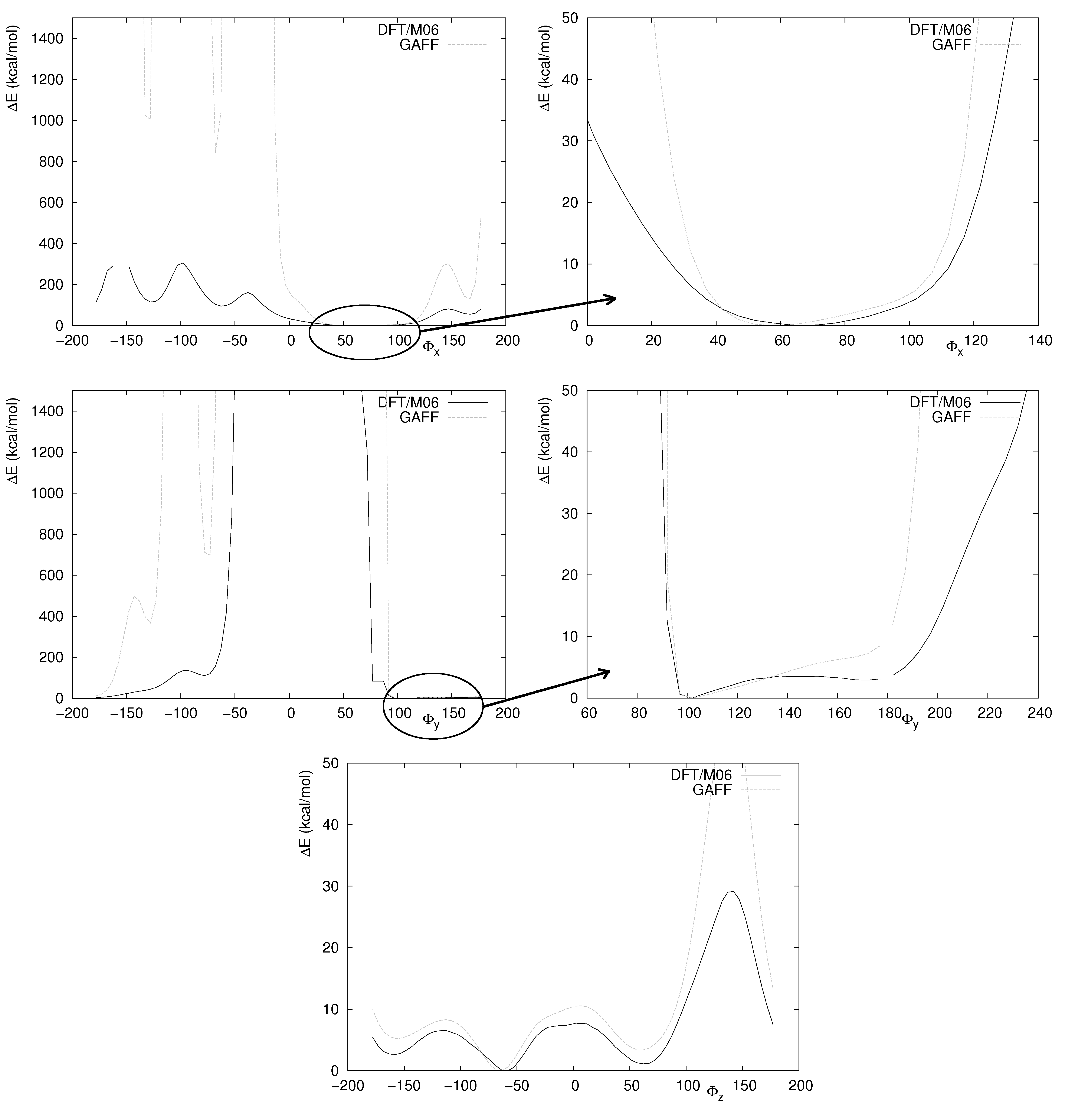


**GOF**

**
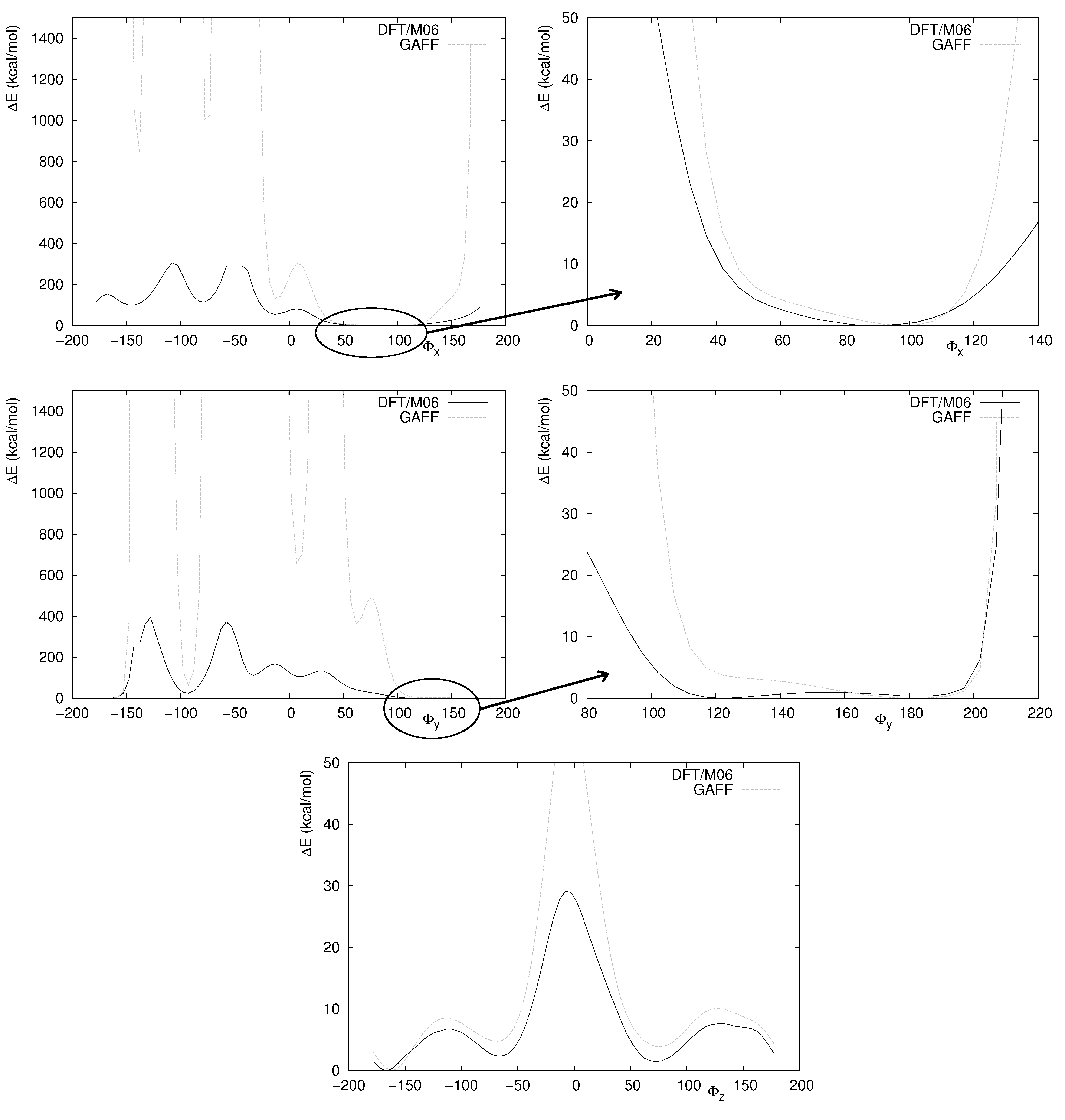
**

**α-S-Galcer**


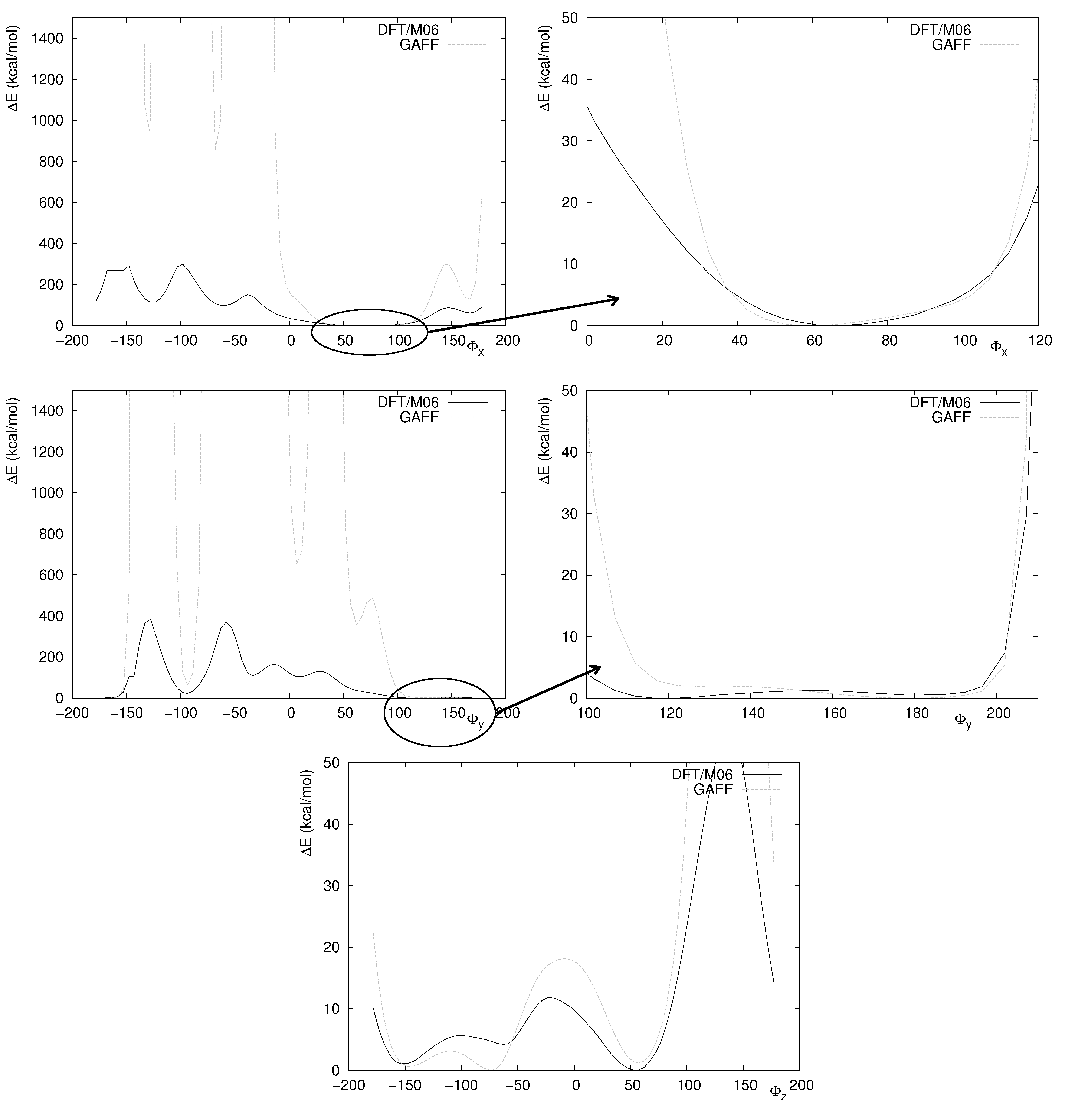


**AZOL**


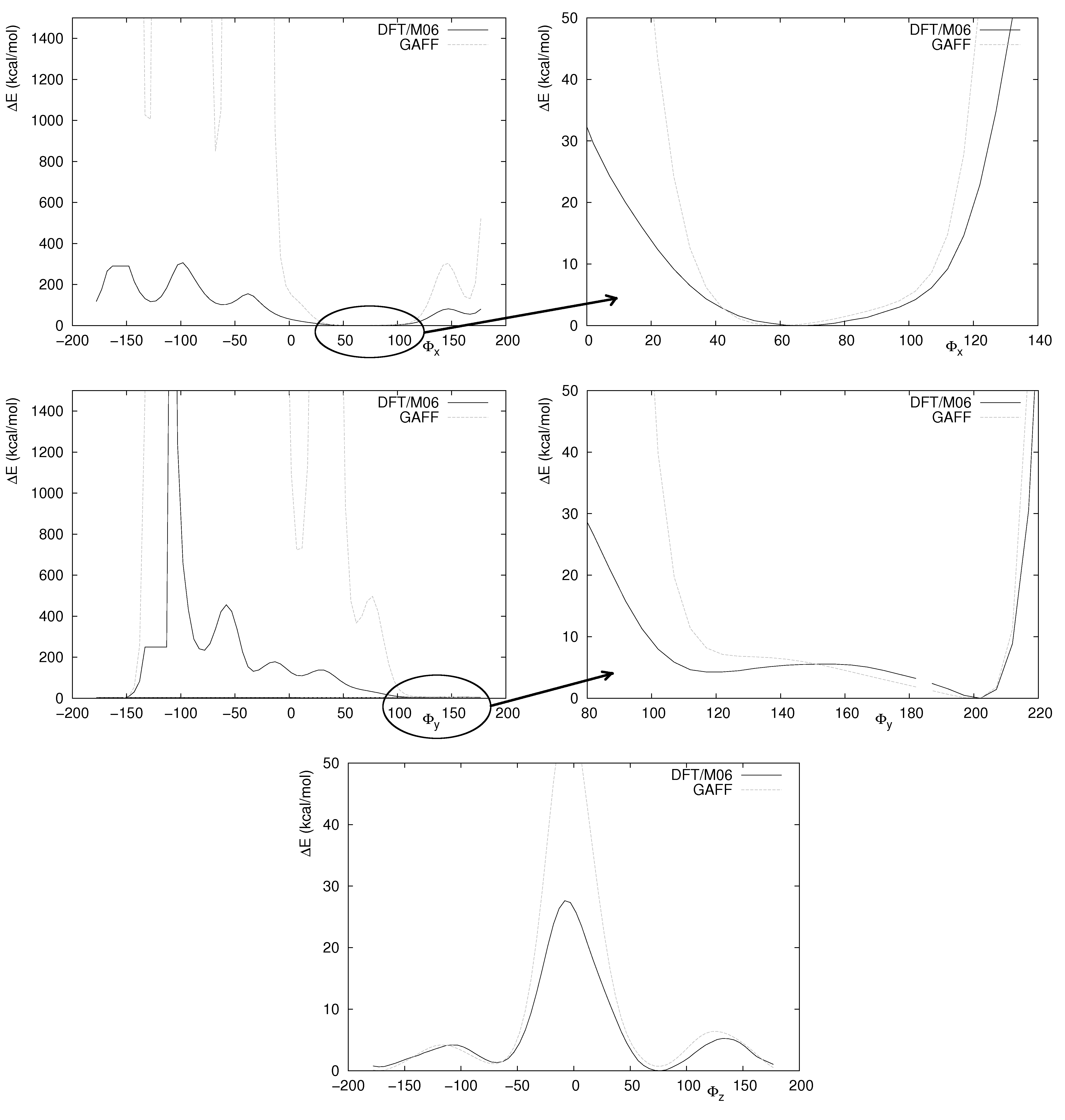

Supplement: Figure S1 — Torsion energy profiles for the polar head of the 8 simulated ligands. The rigid potential energy scans for internal rotations about the three axes φx, φy, φz were performed to compare GAFF force field energies to electronic structure calculations (DFT/6-31G* using the M06 hybrid functional). (DOCX) [file pcbi.1003902.s001.docx]

**
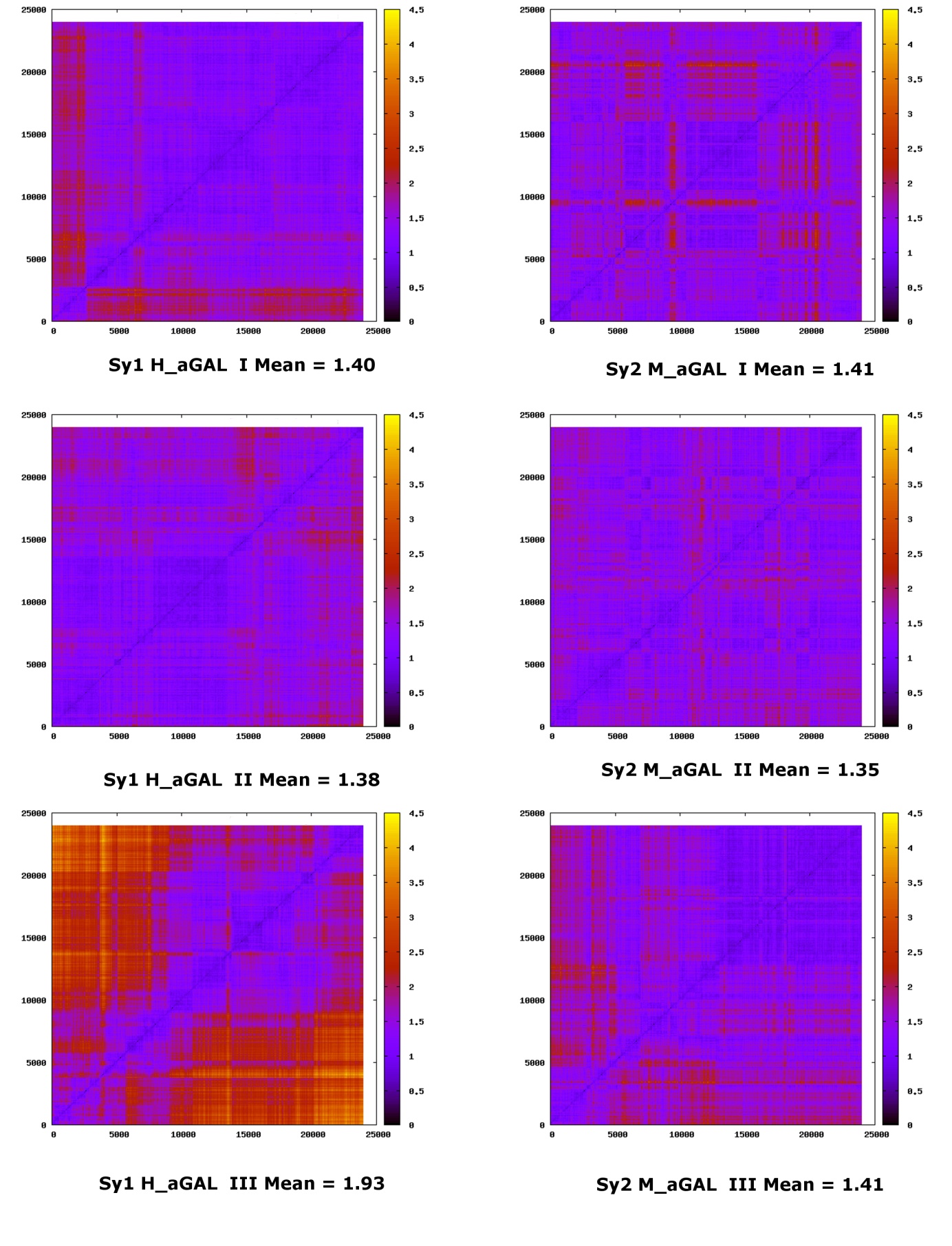
**

**
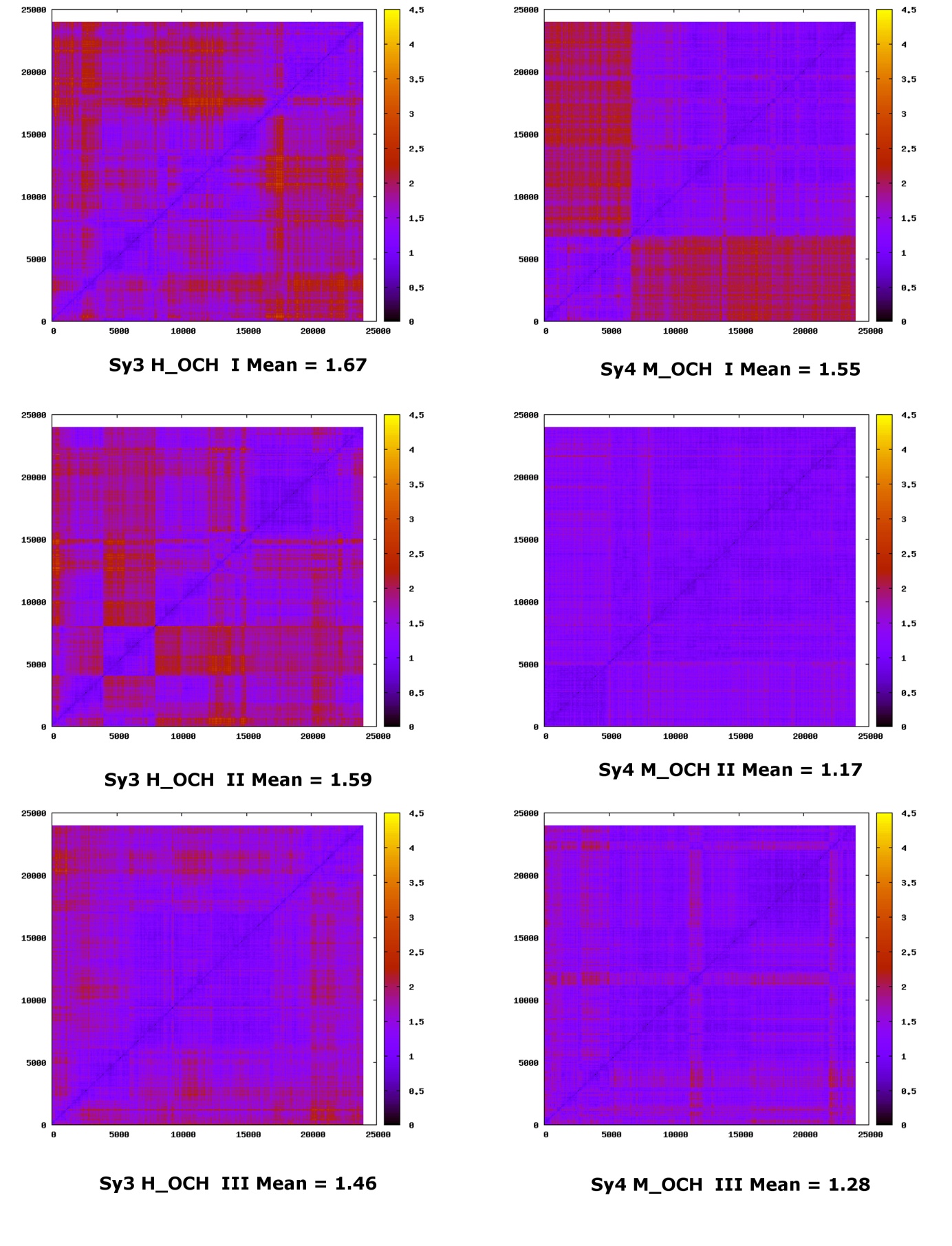
**
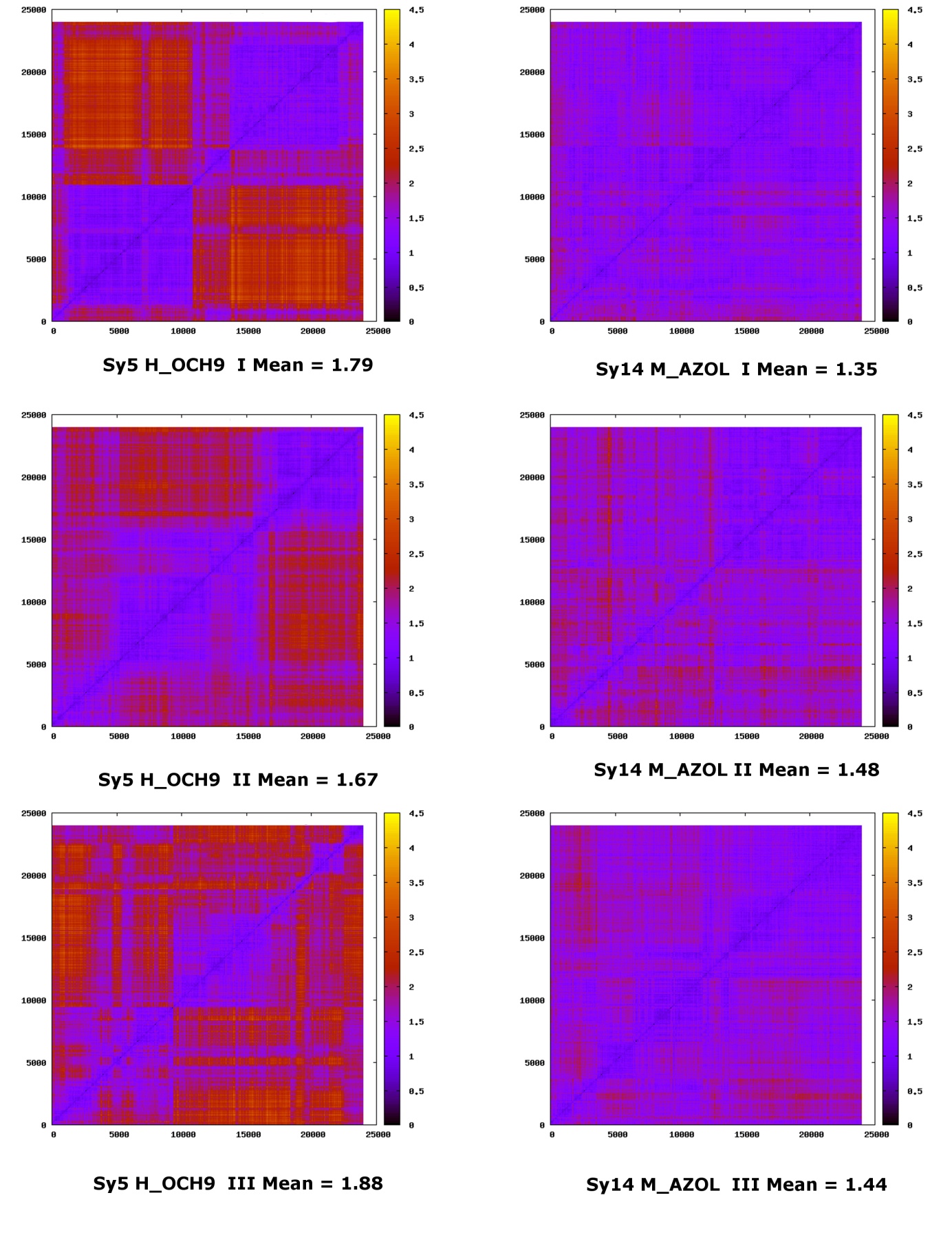


**
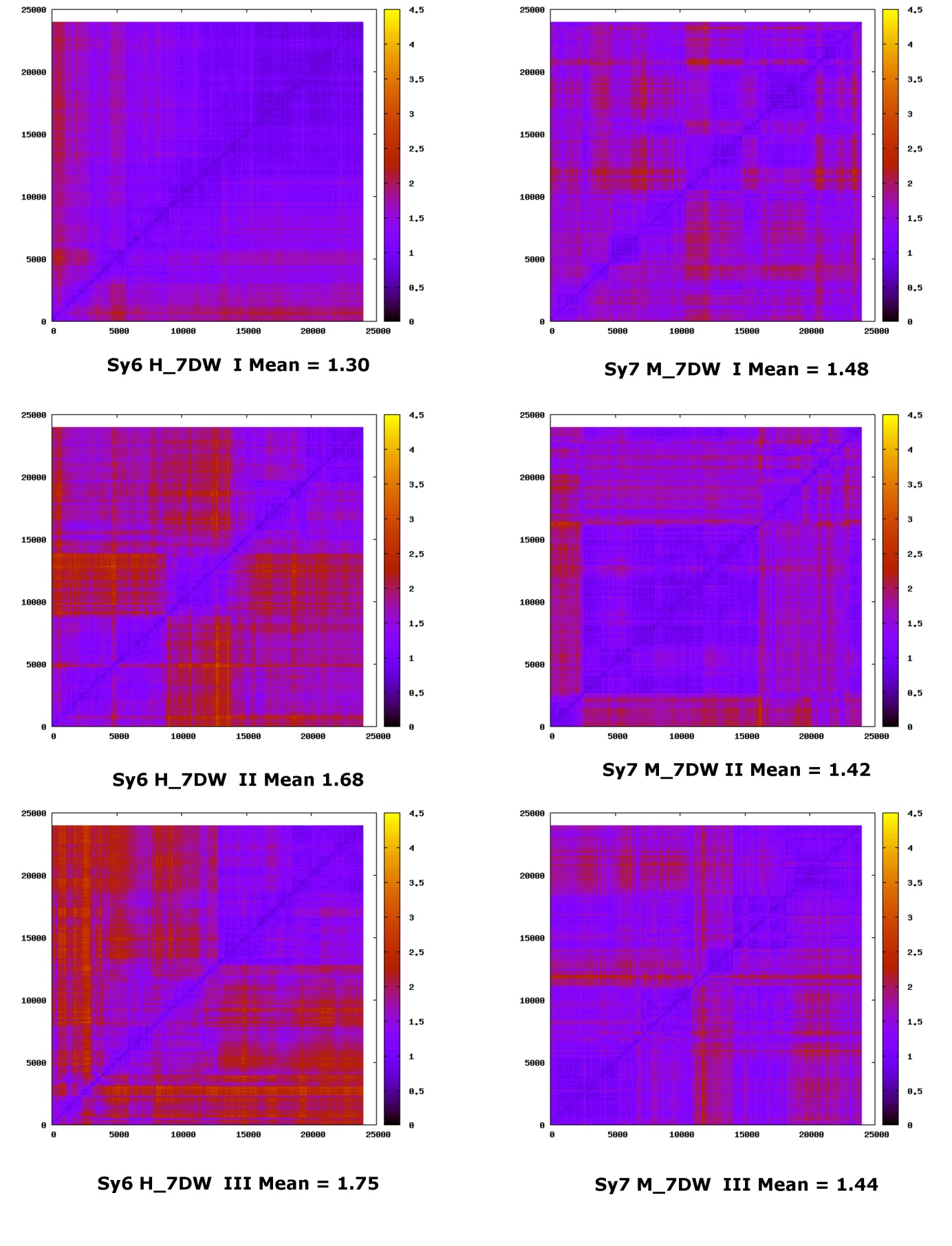
**

**
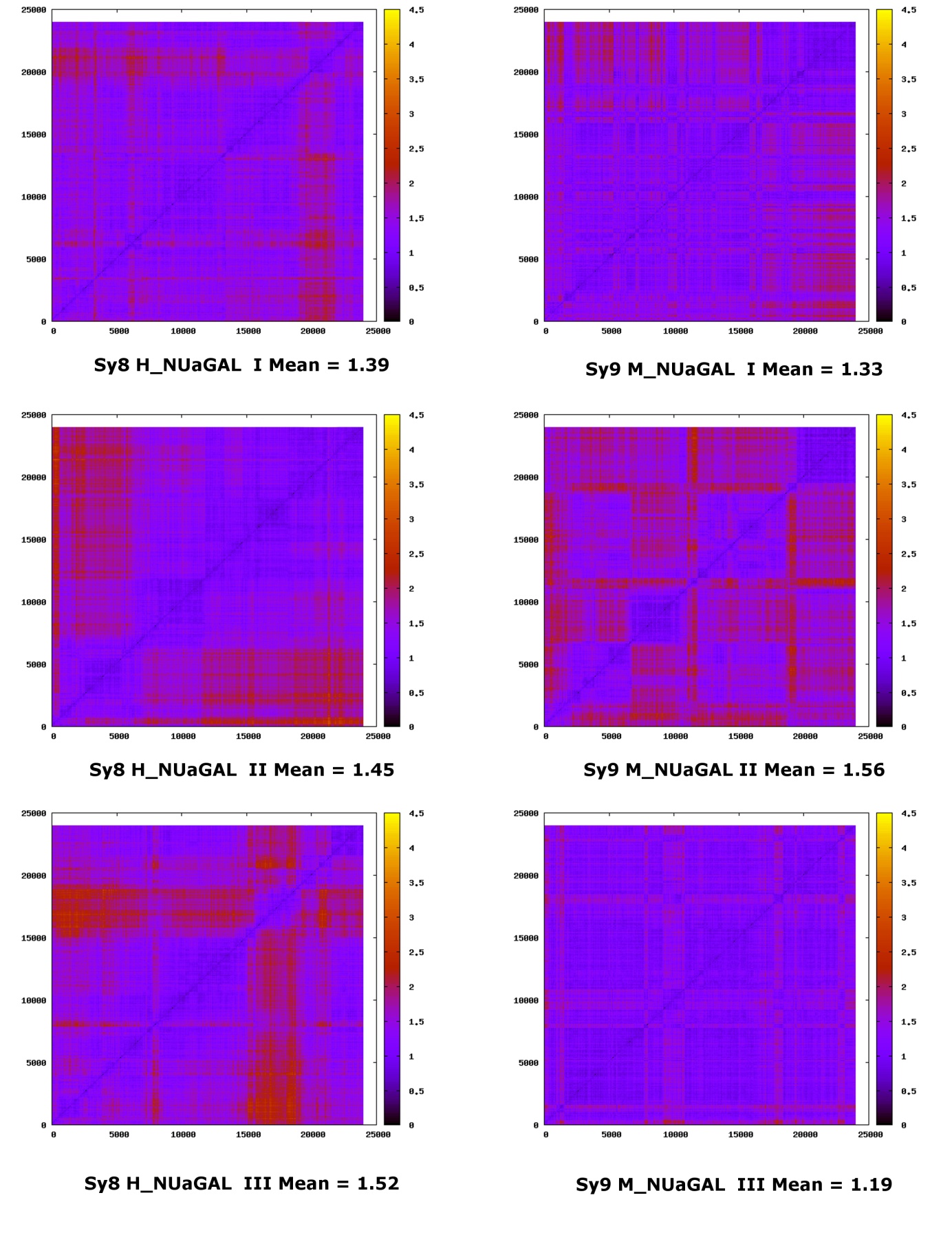
**

**
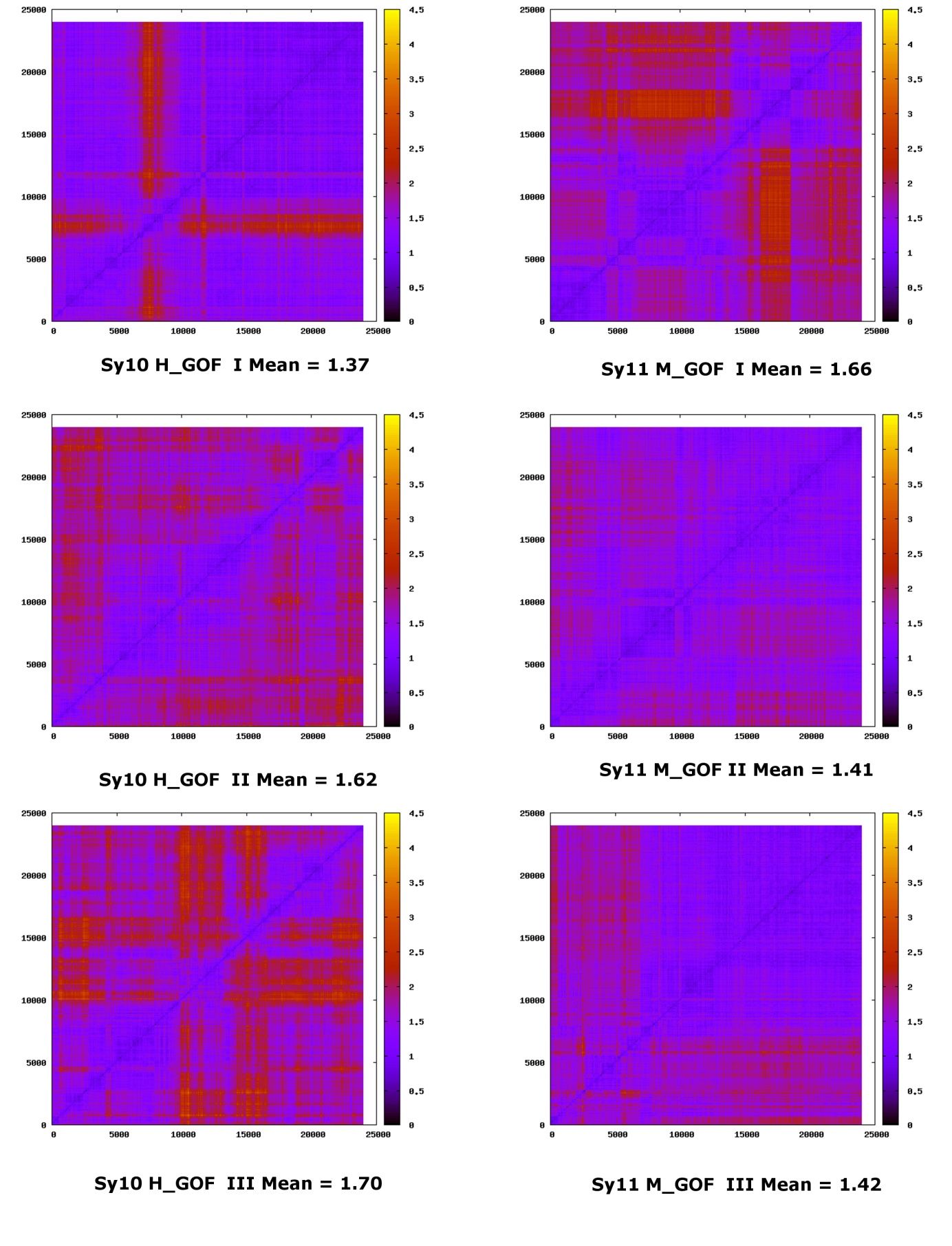
**

**
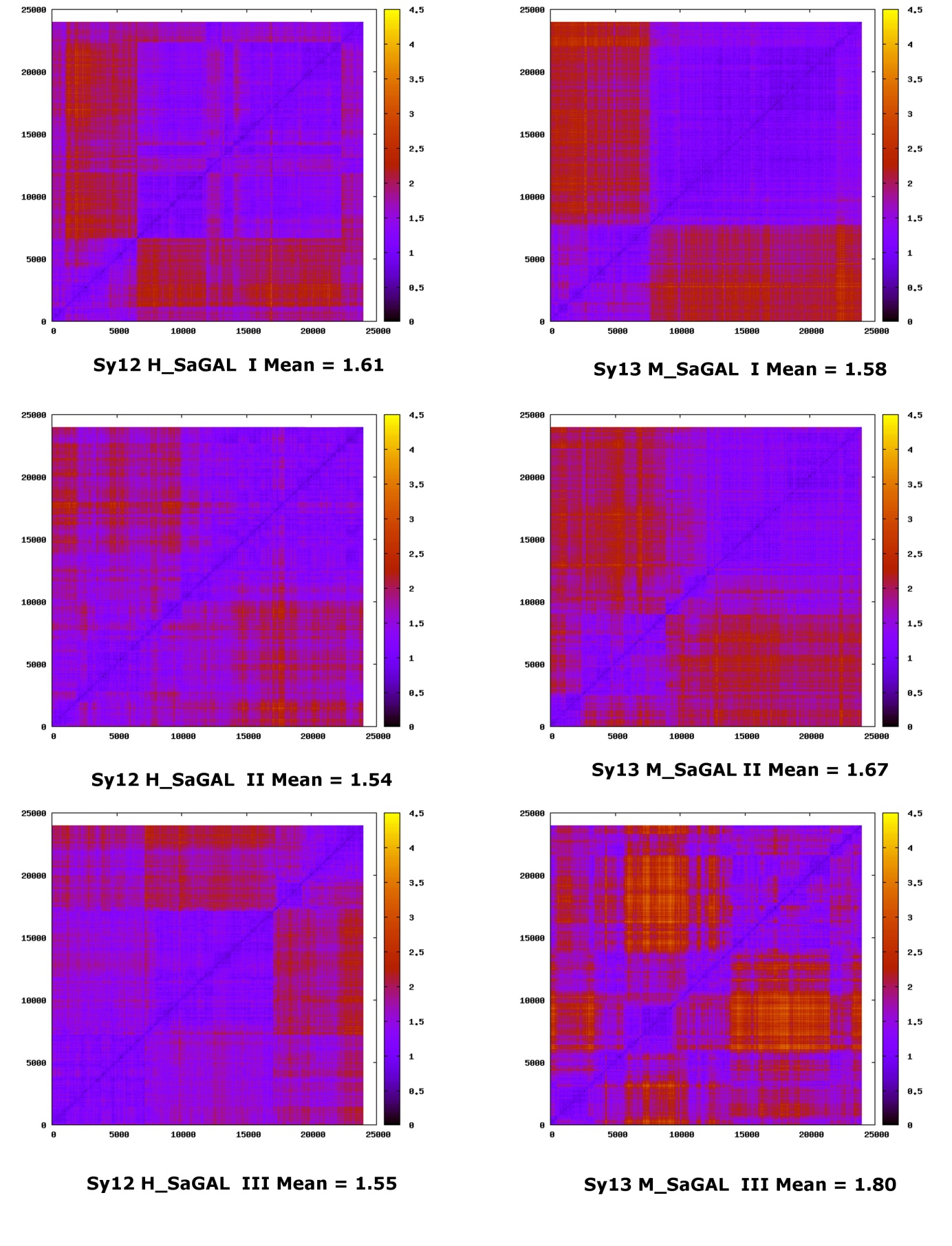
**

**
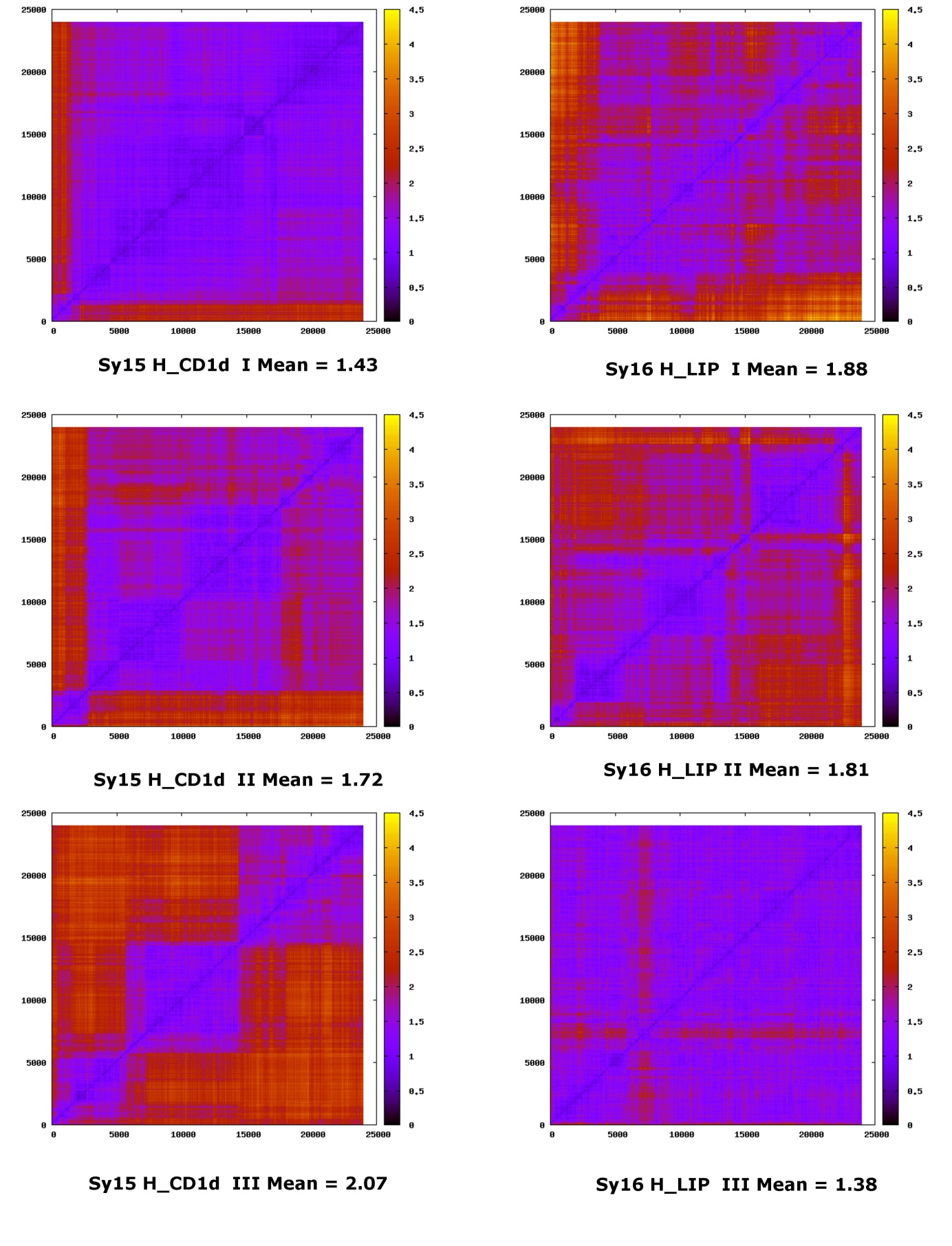
**

Supplement: Figure S2 — 2D-RMSD graphs for all the 16 simulated systems. The average of all RMSD values of this matrix was also computed for each graph. The resulting number represents the level of fluctuation of the whole system during the simulation. (DOCX) [file pcbi.1003902.s002.docx]

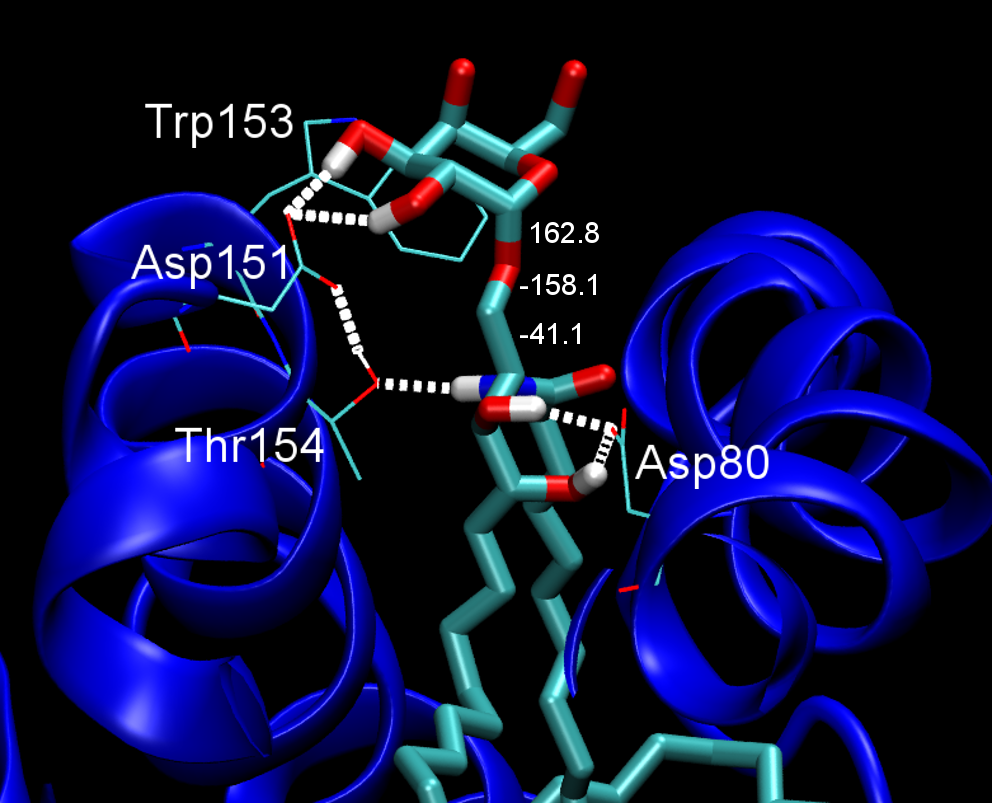

Supplement: Figure S3 — H_aGAL first secondary state. This is a snapshot taken from the simulation of the H_aGAL system (replica I). The conformation of the polar head corresponds to a secondary conformational state different from the main OTAN conformation. But, as can be seen, the polar head is still attached to helix α2. Actually, the combination of the two rotations about φx and φz brings again the polar head in hydrogen contact with residues to helix α2. This state is not specific to the Th1 biological response and can appear in 3D-FEL of Th2 systems such as H_OCH9 or M_AZOL. (TIFF) [file pcbi.1003902.s003.tiff]

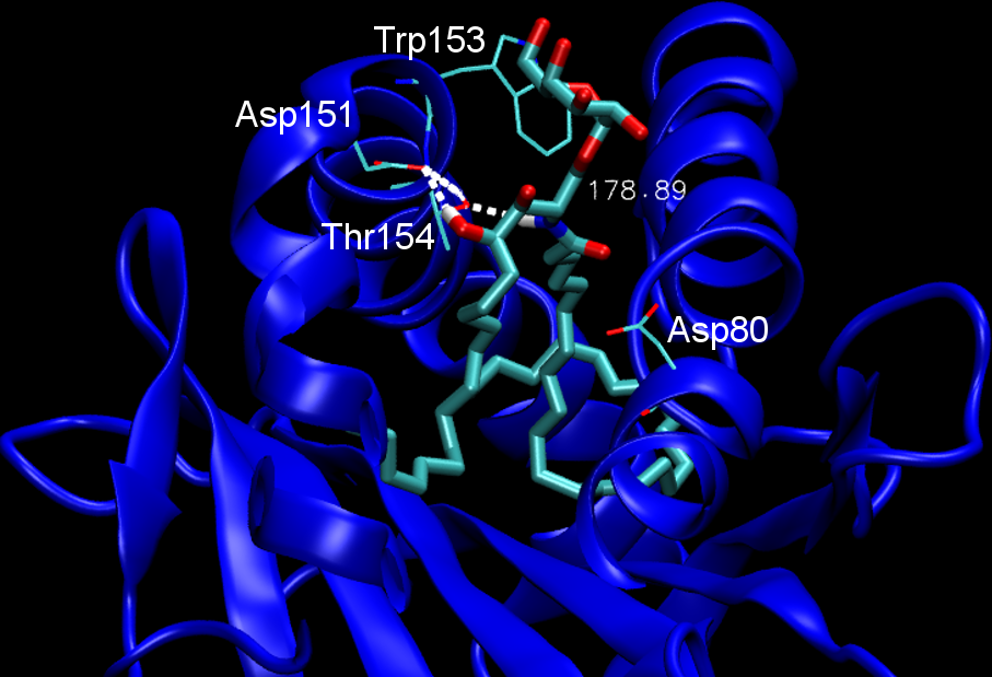

Supplement: Figure S4 — H_aGAL second secondary state. This is a snapshot taken from the simulation of the H_aGAL system (replica III). The conformation of the polar head corresponds to a third conformational state. As can be seen, the polar head is still in contact with helix α2 (VDW contact with Trp153). This state showing a major rotation about φz axis is not specific to the Th1 biological response and can appear in 3D-FEL of Th2 systems such as H_OCH or H_OCH9. (TIFF) [file pcbi.1003902.s004.tiff]

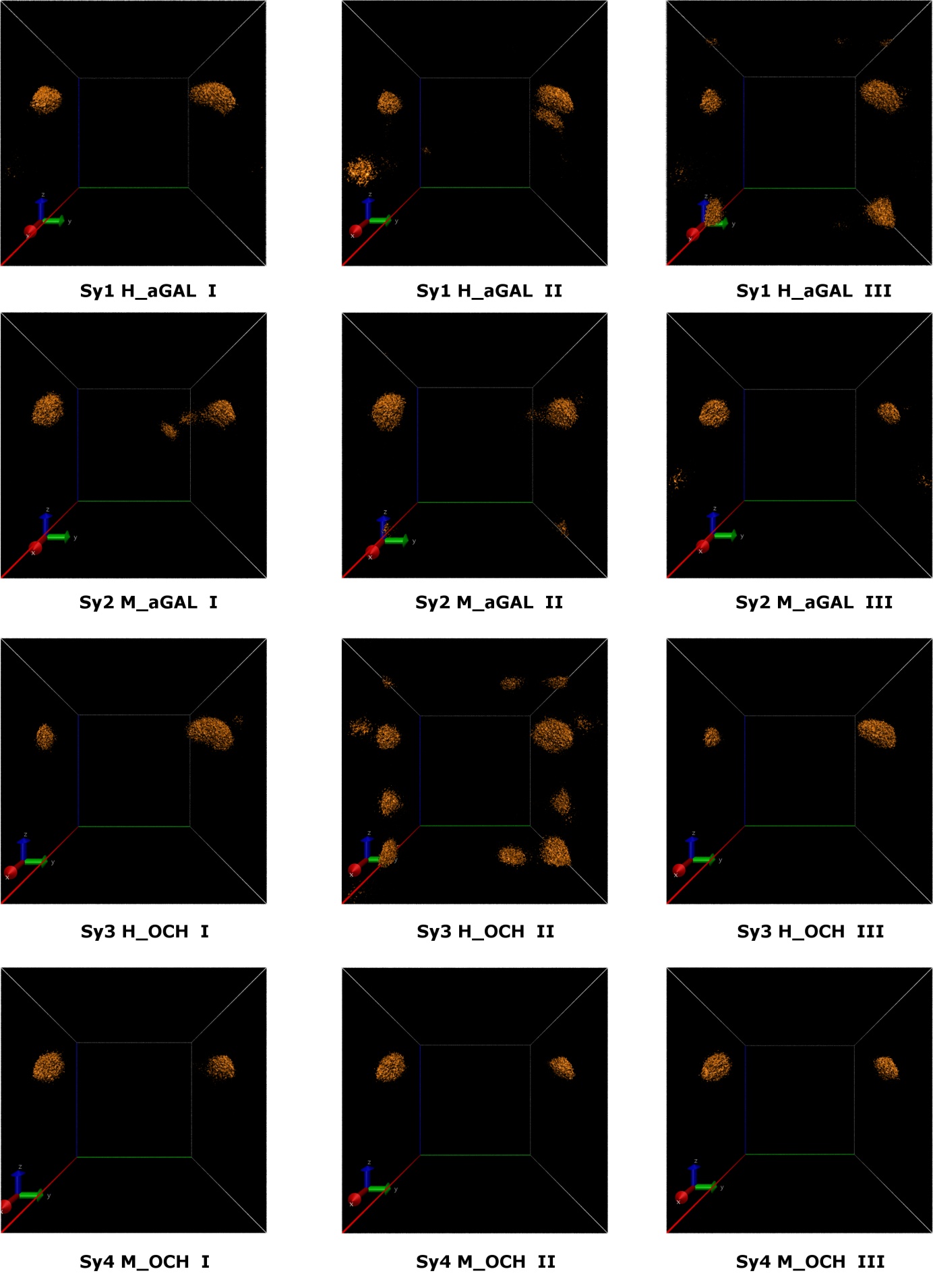


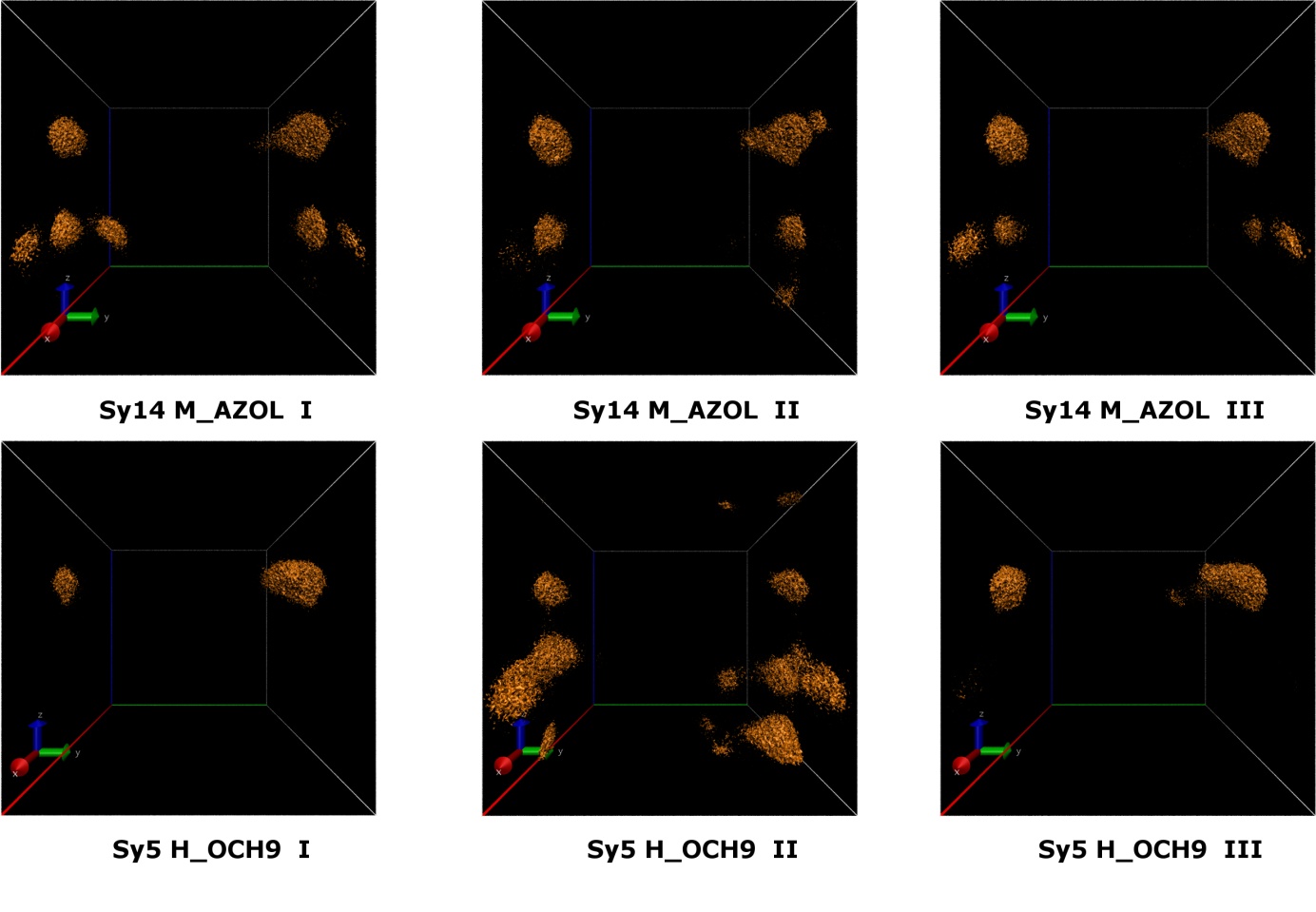


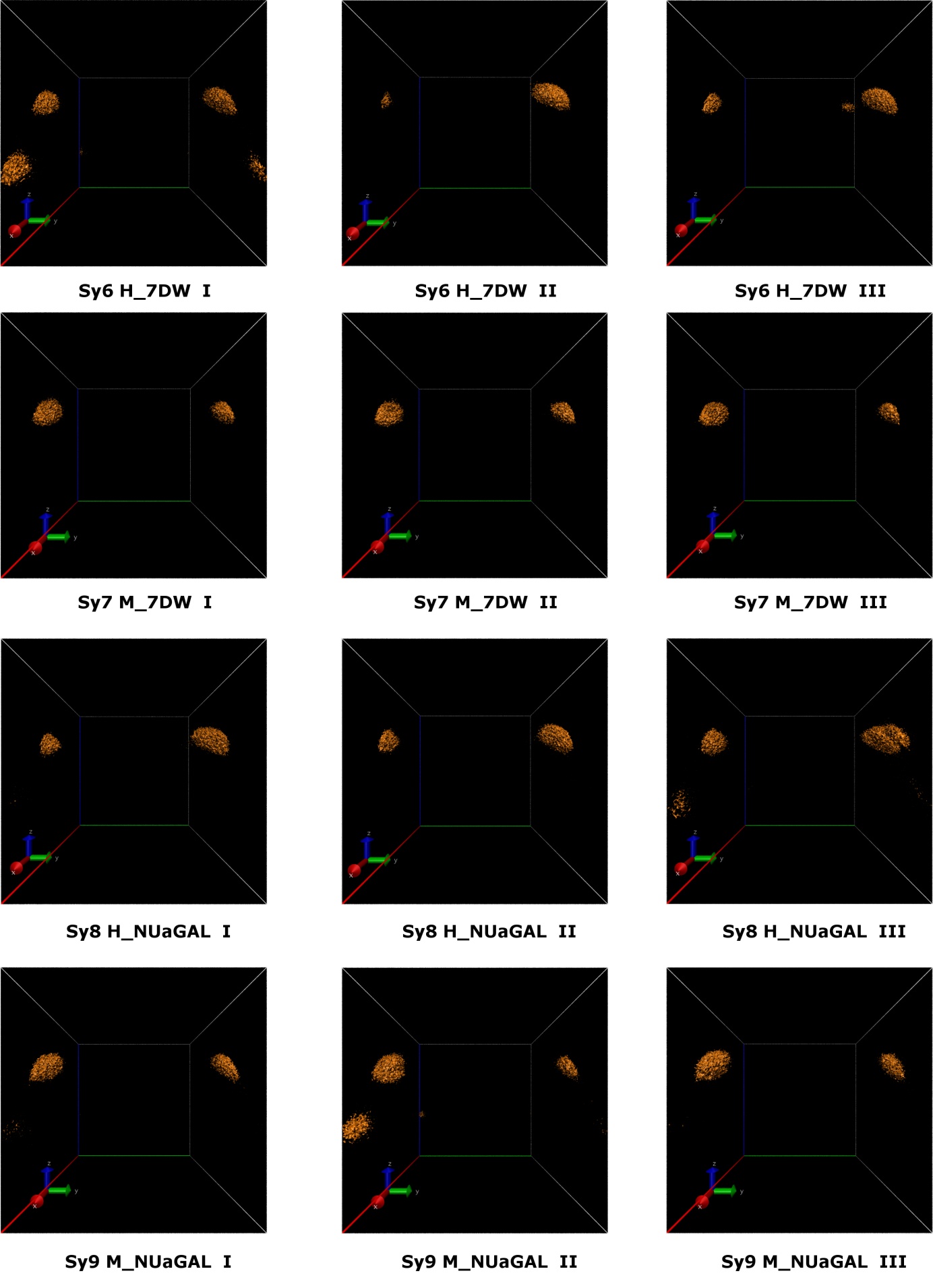


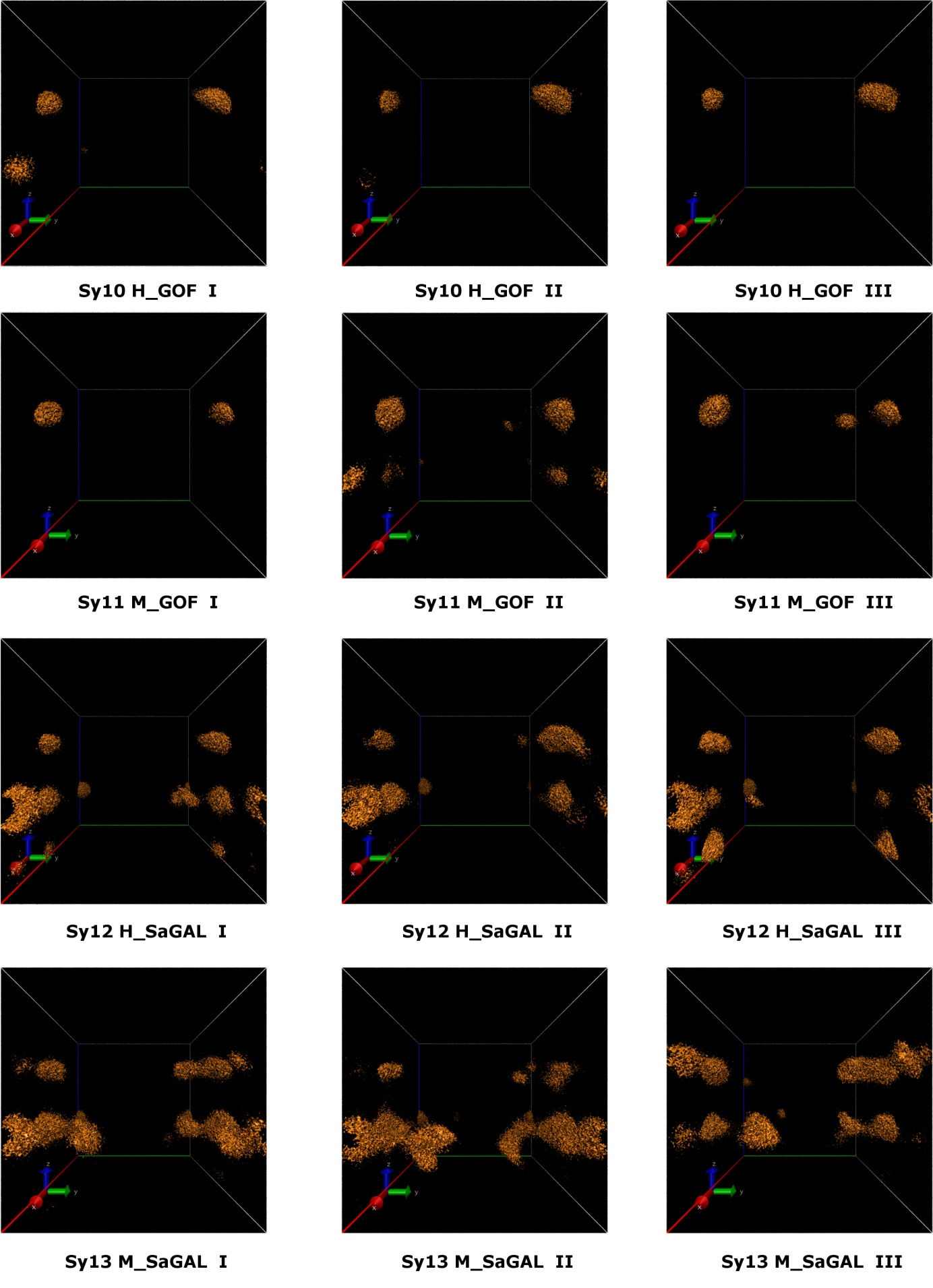

Supplement: Figure S5 — 3D-Free Energy Landscape of all systems at 9 kBT. The conformational space explored by the polar head of the ligand during the simulations was described using the three torsion angles of the three successive rotatable bonds (starting from the anomeric bond). The resulting 3D Free Energy Landscapes are reported below. (DOCX) [file pcbi.1003902.s005.docx]

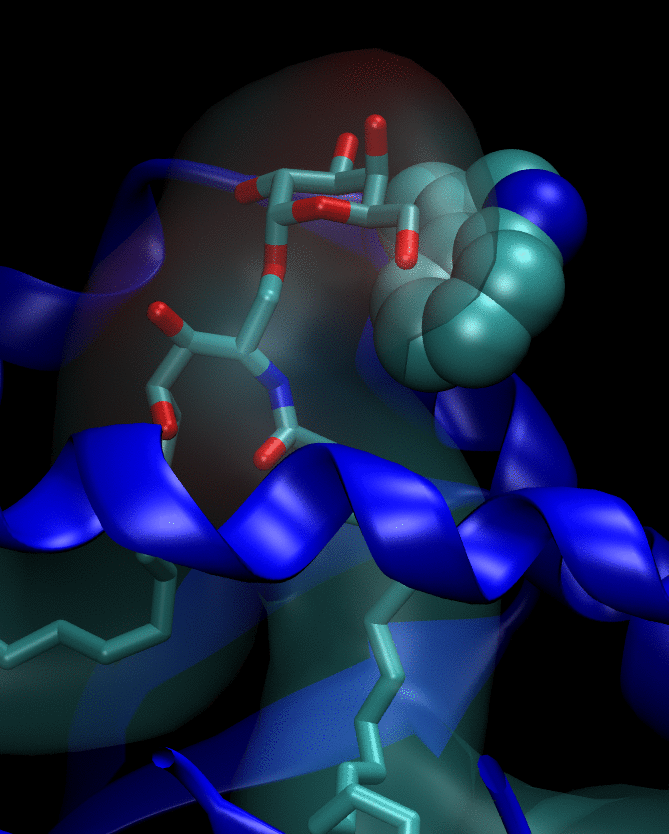

Supplement: Movie S1 — Molecular dynamics simulation (2 ns) of α-Galcer in H_CD1d. This animation was built from the molecular dynamics simulation (sampled every 10 ps) of α-Galcer in human CD1d; it illustrates the van der Waals interaction of residue Trp153 (van der Waals representation) with the hydrophobic part of the sugar head; for the sake of clarity, H atoms and water molecules are not displayed. (GIF) [file pcbi.1003902.s008.gif]
